# Hypoxia-activated DLL4/NOTCH–EDN1 signaling maintains fibroblast activation and endothelial crosstalk in a human dermal fibroblast model of keloid-like remodeling

**DOI:** 10.1186/s41065-026-00717-3

**Published:** 2026-07-29

**Authors:** Shenxi Zhang, Xue Zhang, Xiaoyang Wang

**Affiliations:** Department of Dermatology, Beijing Anzhen Hospital, Capital Medical University, Beijing, Chaoyang 100029 China

**Keywords:** Keloid, Hypoxia, DLL4, NOTCH, EDN1, Fibroblast

## Abstract

To model selected hypoxia-associated features of keloid-like fibroblast remodeling, this study used normal human dermal fibroblasts exposed to hypoxia, with TGF-β1 stimulation used to establish a profibrotic fibroblast phenotype. Hypoxia is a hallmark of keloid tissues and contributes to fibroblast dysfunction and abnormal vascular remodeling. However, the molecular mechanisms linking hypoxia to fibroblast activation and paracrine angiogenesis remain unclear. Here, using normal human dermal fibroblasts as an in vitro approximation of hypoxia-associated keloid-like remodeling, we show that hypoxia activates DLL4/NOTCH and endothelin (EDN1) signaling. Loss-of-function assays showed that DLL4 and EDN1 are required to maintain hypoxia-associated fibroblast proliferation, survival, migration, and fibrotic remodeling. Conditioned medium from hypoxic fibroblasts enhanced endothelial tube formation and migration, whereas these responses were attenuated when conditioned medium was obtained from DLL4- or EDN1-silenced fibroblasts. These findings indicate that fibroblast DLL4/NOTCH and EDN1 signaling influence the hypoxia-conditioned secretome, although the relative contributions of EDN1, VEGF, and other soluble mediators were not resolved. Loss-of-function and pharmacological inhibition experiments showed that full EDN1 expression and secretion under hypoxia depended on DLL4-associated NOTCH activity, with NOTCH1 contributing functionally to this response; however, involvement of other NOTCH receptors was not examined. Recombinant EDN1 partially restored proliferation, migration, and angiogenic support in DLL4-deficient fibroblasts. These findings support functional coupling between DLL4-associated NOTCH and EDN1 signaling but do not establish direct transcriptional regulation of EDN1 by NOTCH. Furthermore, asymmetric functional coupling between DLL4/NOTCH and EDN1 pathways. This study shows that hypoxia induces coordinated DLL4/NOTCH and EDN1 signaling in dermal fibroblasts and that EDN1 mediates part of the DLL4-associated fibroblast activation and endothelial-supportive phenotype.

**Supplementary Information:**

The online version contains supplementary material available at https://doi.org/10.1186/s41065-026-00717-3.

## Introduction

The development of keloids, which are due to abnormalities in the repair process, is not like that of normal wounds; instead, it is the result of sustained inflammation, ongoing activity of fibroblasts, and changes to how signals are processed in the immediate environment of the tissue during healing [1, 2]. While the biological mechanisms that underlie keloid formation have been further elucidated than ever before, there are still very few good treatment options because we still do not completely understand the molecular pathways that connect the activation of fibroblasts to the remodeling of blood vessels [2, 3].

A key characteristic of keloids is the concurrent occurrence of hypoxia within keloidal tissues resulting from a selective buildup of ECM components and the abnormal arrangement of blood vessels that decrease their ability to carry oxygen to the tissue [4, 5]. In this situation, hypoxia causes the stabilization of HIF-1α (a transcriptional regulator) that controls the output of genes regulating angiogenesis, metabolic responses and/or survival of cells, such as VEGFA [6]. Additionally, in keloids, increased vascular density is linked with increased amounts of HIF-1α and VEGF indicating a role for hypoxia in regulating both fibrotic and angiogenic processes [7]. However, the mechanism(s) by which hypoxia promotes fibroblast activation and provides paracrine signals for angiogenesis are not yet fully understood.

The DLL4-mediated Notch signaling pathway regulates cell fate determination and angiogenesis development [8]. DLL4/NOTCH signaling (as a modulator of endothelial VEGF mediated tip cancer cell to nest cell differentiation and subsequent vascular tubing) is also shown to be involved with fibroblasts via regulation of proliferation, differentiation, and fibrosis [9]. Notably, hypoxic conditions enhance Notch activation by HIF-1α, linking oxygen sensing to cell fate decisions [10, 11]. There is limited research performed examining the role of DLL4 / NOTCH within keloid fibroblasts and the contributions of DLL4/NOTCH signaling towards integrating fibrotic and hypoxic responses. The endothelin system (notably EDN1) has been linked to angiogenesis to both fibrotic processes and endothelial dysfunction. EDN1 is a strong vasoactive peptide that can stimulate vasoconstriction, cell proliferation, and ECM deposition by activation of endothelin receptors (EDNRA and EDNRB) [12]. EDN1 encourages myofibroblasts to become active and make collagen in fibrotic disorders, usually in combination with TGF-β [12, 13]. Several studies report that keloid fibroblasts express high levels of EDN1 compared to normal human fibroblasts having this peptide secretion and that it has an important role in both activating fibroblasts and producing abnormal blood vessel patterns [12, 14]. As such, further research is needed to define the upstream regulators of Edn1 in keloid fibroblasts, specifically under hypoxia, and to determine how other cellular signalling pathways interact with EDN1. There is limited research performed examining the role of DLL4 / NOTCH within keloid fibroblasts and the contributions of DLL4/NOTCH signaling towards integrating fibrotic and hypoxic responses. Because DLL4 can engage more than one NOTCH receptor, the present study examined NOTCH1 as a functionally relevant receptor but was not designed to establish receptor exclusivity.

Notch and endothelin signaling pathways have been shown to potentially interact with one another but the exact nature of this interaction between these two pathways is determined by the context in which they are activated as their relationship has not yet been fully determined. Notch signaling has been demonstrated to regulate the expression of many vasoactive and angiogenic factors; conversely, EDN1 can affect many intracellular signalling pathways, some of which develop the same changes in their downstream pathways as Notch does [15, 16]. Additionally, both signalling pathways converge at several key aspects of cell behaviour including proliferation, survival, and the remodelling of the extracellular matrix, thus providing the potential for a coordinated regulatory network between these two signalling pathways in the context of fibrotic disease pathophysiology. However, the existence of crosstalk between both Notch and endothelin signalling pathways in keloid fibroblasts and whether this crosstalk, if it does exist, contributes to hypoxia-induced pathology has yet to be thoroughly investigated. In addition to these cell-autonomous effects, fibroblasts also play an important function in forming a supportive microenvironment within the tissue through paracrine signalling and determining how endothelial cells behave and form new blood vessels [17, 18]. The factors produced by fibroblasts in keloids, promote endothelial cell proliferation, migration, and tube formation, leading to aberrant blood vessel formation [19]. As both Notch and endothelin signalling pathways independently regulate the process of angiogenesis, it is reasonable to speculate that both hypoxia-activated fibroblasts may modulate endothelial cell function through pathways dependent on DLL4/NOTCH- and EDN1, however, limited evidence currently exists supporting this hypothesis.

Although hypoxia, NOTCH/DLL4 signaling, and endothelin signaling have each been implicated previously in fibrosis, angiogenesis, and wound-associated remodeling, their functional relationship in keloid fibroblasts remains insufficiently defined. Therefore, the central question is not simply whether hypoxia can induce these established mediators, but whether their hypoxic activation represents a functionally connected signaling relationship that contributes to fibroblast activation and paracrine endothelial modulation. In particular, it remains unclear whether DLL4/NOTCH activity is required for full EDN1 expression in hypoxic fibroblasts, whether EDN1 mediates part of the DLL4-dependent fibroblast phenotype, and whether this interaction has practical relevance for targeting both fibrotic remodeling and aberrant angiogenic crosstalk in keloid disease.

Because this study did not use primary keloid-derived fibroblasts or keloid tissue specimens, normal human dermal fibroblasts exposed to hypoxia and TGF-β1 were used as a reductionist in vitro model to approximate selected features of keloid-like fibroblast remodeling. Hypoxia was selected because oxygen deprivation is a recognized feature of fibrotic scar microenvironments, while TGF-β1 stimulation was used to induce a profibrotic fibroblast phenotype characterized by increased extracellular matrix and myofibroblast-associated marker expression. This model does not fully reproduce the cellular heterogeneity or disease-specific memory of keloid lesions, but it allows controlled evaluation of hypoxia-responsive signaling interactions that may contribute to fibroblast activation and endothelial crosstalk.

In this study, we investigated whether hypoxia-associated DLL4/NOTCH and EDN1 signaling are functionally connected in normal human dermal fibroblasts and whether this interaction is required for maintaining fibroblast activation and endothelial angiogenic support in an in vitro model of keloid-like remodeling. Using siRNA-mediated knockdown, conditioned-medium assays, luciferase reporter analysis, EDN1 secretion measurement, and recombinant EDN1 rescue experiments, we assessed the relative contribution of DLL4/NOTCH and EDN1 signaling under hypoxic conditions. The results support an asymmetric bidirectional DLL4/NOTCH–EDN1 signaling relationship that may represent a candidate mechanism relevant to hypoxia-associated fibroblast activation in keloid disease.

## Materials and methods

### Cell lines and culture conditions

Human dermal fibroblasts (HDFs; ATCC PCS-201-012) were cultured in Dulbecco’s Modified Eagle Medium (DMEM; Thermo Fisher Scientific, Cat. No. 11965092) supplemented with 10% heat-inactivated fetal bovine serum (FBS; Gibco, Cat. No. 10099141 C) and 1% penicillin–streptomycin (Gibco, Cat. No. 15140122). Human dermal microvascular endothelial cells (HDMECs; Lonza, Cat. No. CC-2543) were maintained in EGM-2 MV medium (Lonza, Cat. No. CC-3202) containing manufacturer-supplied growth supplements. Cells were incubated at 37 °C in a humidified atmosphere with 5% CO₂ and passaged at 70–80% confluence using 0.05% trypsin-EDTA (Gibco, Cat. No. 25300054). HDFs were normal human dermal fibroblasts and were not derived from keloid lesions. Therefore, all fibroblast experiments should be interpreted as an in vitro model of hypoxia- and TGF-β1-associated keloid-like remodeling rather than as direct analysis of primary keloid fibroblasts. All experiments were performed using cells between passages 3 and 8.

### TGF-β1-induced fibroblast activation

To establish a profibrotic fibroblast phenotype, HDFs were treated with recombinant human transforming growth factor-beta 1 (TGF-β1; Thermo Fisher Scientific, Cat. No. PHG9214) at a concentration of 10 ng/mL for 48 h prior to downstream analyses. Control cells were cultured under identical conditions without TGF-β1 supplementation.

### Hypoxia exposure

For hypoxia experiments, cells were exposed to a low-oxygen environment (1% O₂, 5% CO₂, 94% N₂) using a Biospherix Xvivo hypoxia chamber. Cells were seeded at appropriate densities and allowed to adhere overnight under normoxic conditions before transfer to the hypoxia chamber. Hypoxic exposure was conducted for 24 h unless otherwise indicated. Parallel control cultures were maintained under normoxic conditions (21% O₂). Culture media were pre-equilibrated under hypoxic conditions prior to use to minimize oxygen fluctuations.

### siRNA-mediated gene silencing

Gene silencing was performed using two independent non-overlapping Silencer™ Select siRNAs (Thermo Fisher Scientific, Cat. No. 4392420) for each target: DLL4 (Assay IDs s21106 and s21105), NOTCH1 (s9633 and s9634), and EDN1 (s4817 and s4818). A non-targeting Silencer™ Select Negative Control No. 1 siRNA (Cat. No. 4390843) was used as the control. HDFs at 50–60% confluence was transfected with 30 nM siRNA using Lipofectamine™ RNAiMAX (Thermo Fisher Scientific, Cat. No. 13778150) in Opti-MEM™ medium (Gibco, Cat. No. 31985070). After 6 h, the medium was replaced with complete DMEM, and cells were cultured for 48 h before analysis. For dual knockdown, DLL4 and EDN1 siRNAs were co-transfected at 15 nM each. Knockdown efficiency was confirmed by RT-qPCR and Western blotting. Key pathway and functional findings were independently reproduced using the second siRNA for each gene to minimize sequence-specific off-target effects.

### Recombinant EDN1 rescue experiments

To assess whether EDN1 acts downstream of DLL4 signaling, rescue experiments were performed using recombinant human endothelin-1 (EDN1; R&D Systems, Cat. No. 1160-ET). Following siRNA-mediated DLL4 knockdown, fibroblasts were exposed to hypoxic conditions and treated with recombinant EDN1 at a final concentration of 50 nM for 24 h prior to functional assays. Control groups received vehicle treatment. Cells and conditioned media were subsequently collected for proliferation, migration, and endothelial functional assays.

### RNA isolation and RT-qPCR

Total RNA was extracted using TRIzol™ Reagent (Thermo Fisher Scientific, Cat. No. 15596026) according to the manufacturer’s instructions. RNA concentration and purity were assessed using a NanoDrop 2000 spectrophotometer (Thermo Fisher Scientific). cDNA synthesis was performed using the High-Capacity cDNA Reverse Transcription Kit (Thermo Fisher Scientific, Cat. No. 4368814) with 1 µg of total RNA in a 20 µL reaction. Quantitative real-time PCR was carried out using TaqMan™ Fast Advanced Master Mix (Thermo Fisher Scientific, Cat. No. 4444557) on a QuantStudio™ 6 Flex Real-Time PCR System. The following TaqMan Gene Expression Assays were used: DLL4 (Hs00184092_m1), NOTCH1 (Hs01062014_m1), HES1 (Hs00172878_m1), HEY1 (Hs01114113_m1), EDN1 (Hs00174961_m1), EDNRA (Hs00240747_m1), EDNRB (Hs00240743_m1), HIF1A (Hs00153153_m1), VEGFA (Hs00900055_m1), COL1A1 (Hs00164004_m1), ACTA2 (Hs00426835_g1), FN1 (Hs01549976_m1), and GAPDH (Hs02786624_g1). Relative gene expression was calculated using the 2^^−ΔΔCt^ method with GAPDH as the internal control. For endothelial validation experiments, HDMECs treated with fibroblast-conditioned medium were harvested for RNA extraction and analyzed using the same protocol. To specifically assess endothelial activation and angiogenic response, the expression of angiogenesis-related genes, including VEGFA, DLL4, NOTCH1, HES1, and HEY1, was evaluated. Relative gene expression was calculated using the 2^^−ΔΔCt^ method with GAPDH as the internal control.

### Protein extraction and Western blot analysis

Cells were lysed in ice-cold RIPA buffer (Thermo Fisher Scientific, Cat. No. 89900) supplemented with protease and phosphatase inhibitor cocktails (Thermo Fisher Scientific, Cat. No. 78442). Protein concentrations were determined using the Pierce BCA Protein Assay Kit (Thermo Fisher Scientific, Cat. No. 23225). Equal amounts of protein (20–30 µg) were separated by SDS-PAGE and transferred onto PVDF membranes (Millipore, Cat. No. IPVH00010). Membranes were blocked with 5% non-fat milk in TBST and incubated overnight at 4 °C with primary antibodies against DLL4 (Abcam, Cat. No. ab7280), NOTCH1 (D1E11; Cell Signaling Technology, Cat. No. 3608; 1:1,000), which detects full-length NOTCH1 at approximately 300 kDa and the transmembrane/intracellular NOTCH1 form (NOTCH1 NTM) at approximately 120 kDa but is not cleavage-specific for activated NICD1, HES1 (Cell Signaling Technology, Cat. No. 11988), HIF-1α (Cell Signaling Technology, Cat. No. 36169), EDN1 (Abcam, Cat. No. ab2786), α-SMA (Abcam, Cat. No. ab5694), cleaved caspase-3 (Cell Signaling Technology, Cat. No. 9661), β-actin (Cell Signaling Technology, Cat. No. 4970), and GAPDH (Cell Signaling Technology, Cat. No. 5174). Following incubation with HRP-conjugated secondary antibodies, protein bands were visualized using enhanced chemiluminescence (ECL; Thermo Fisher Scientific, Cat. No. 32106) and imaged using a ChemiDoc imaging system (Bio-Rad). Densitometric analysis was performed using ImageJ software. Protein expression levels were normalized to β-actin and GAPDH. Whole-cell NOTCH1 NTM abundance and HES1 expression were assessed in the initial experiments, whereas activation-specific canonical NOTCH signaling was evaluated separately by quantifying cleaved NICD1 in nuclear fractions following DAPT treatment.

### Immunofluorescence staining

Fibroblasts were fixed with 4% paraformaldehyde, permeabilized with 0.1% Triton X-100, and blocked with 5% bovine serum albumin. Cells were incubated overnight at 4 °C with an α-SMA antibody (Abcam, Cat. No. ab5694), followed by an Alexa Fluor-conjugated secondary antibody and DAPI nuclear counterstaining. Images were acquired at higher magnification using identical exposure and acquisition settings for all groups. Five randomly selected non-overlapping fields were analyzed per biological replicate. α-SMA fluorescence was quantified in ImageJ after background subtraction and normalized to the number of DAPI-positive nuclei.

### Cell proliferation and EdU incorporation assays

Cell proliferation was assessed using the Cell Counting Kit-8 (CCK-8; Dojindo, Cat. No. CK04). Cells were seeded in 96-well plates at a density of 3 × 10³ cells per well and treated as indicated. At designated time points, 10 µL of CCK-8 reagent was added to each well and incubated for 2 h at 37 °C. Absorbance was measured at 450 nm using a microplate reader. DNA synthesis was evaluated using the Click-iT™ EdU Imaging Kit (Thermo Fisher Scientific, Cat. No. C10337) according to the manufacturer’s instructions.

### Colony formation assay

For clonogenic assays, cells were seeded in 6-well plates at a density of 500 cells per well and cultured for 10–14 days. Colonies were fixed with 4% paraformaldehyde, stained with 0.5% crystal violet, and counted under a microscope.

### Apoptosis and cell cycle analysis

Apoptosis was assessed using the Annexin V-FITC Apoptosis Detection Kit (BD Biosciences, Cat. No. 556547) and analyzed using a BD FACSCanto II flow cytometer. At least 10,000 events were acquired per sample. Debris was excluded using forward- and side-scatter gating, followed by singlet selection using FSC-A and FSC-H. Fluorescence compensation and quadrant boundaries were established using unstained and single-stained controls. Cells were classified as viable, early apoptotic, late apoptotic, or necrotic based on Annexin V and PI staining. Total apoptosis was calculated as the sum of early and late apoptotic populations. For cell-cycle analysis, cells were fixed in 70% ethanol, treated with RNase A, stained with PI, and analyzed by flow cytometry. Debris and doublets were excluded using scatter and pulse-geometry gating, and the proportions of cells in G0/G1, S, and G2/M phases were determined using FlowJo software. Identical gating settings were applied across samples within each experiment.

### Wound healing assay

Cells were seeded in 6-well plates and grown to confluence. A scratch was created using a sterile 200 µL pipette tip, and images were captured at 0 and 24 h. Wound closure was quantified using ImageJ software.

### Proliferation-controlled migration assays

To minimize the contribution of cell proliferation, transfected fibroblasts were treated with mitomycin C at a prevalidated non-cytotoxic concentration for 2 h, washed with PBS, and subjected to wound-healing and Transwell migration assays. Wound closure was assessed in serum-reduced medium over 24 h, whereas Transwell migration was evaluated using equal numbers of viable cells. Suppression of proliferation without loss of viability was confirmed by EdU incorporation and trypan-blue exclusion.

### Transwell migration and invasion assays

Migration assays were performed using Transwell inserts (Corning, Cat. No. 3422). Cells were seeded in serum-free medium in the upper chamber, with complete medium in the lower chamber. After 24 h, migrated cells were fixed, stained, and counted. For invasion assays, inserts were pre-coated with Matrigel (Corning, Cat. No. 356234). For endothelial migration assays, HDMECs were treated with fibroblast-conditioned medium and analyzed using the same Transwell protocol.

### Conditioned medium and endothelial tube formation assay

Conditioned medium (CM) was collected from fibroblasts after the indicated siRNA transfection and normoxic or hypoxic treatment. Before CM collection, fibroblasts were washed twice with PBS and cultured for 24 h in serum-reduced DMEM containing 1% FBS under the corresponding oxygen condition. At the time of collection, donor fibroblast viability was assessed by trypan blue exclusion, and CM was normalized to the number of viable donor cells before application to endothelial cells. Specifically, CM was adjusted to an equivalent viable-cell input across groups by dilution with fresh serum-reduced medium, thereby minimizing differences caused by reduced donor-cell survival after DLL4 or EDN1 knockdown. CM was centrifuged at 300 × g for 5 min and then at 2,000 × g for 10 min to remove cells and debris. HDMECs were seeded onto Matrigel-coated plates and treated with normalized CM. Tube formation was imaged after 6–8 h and quantified using ImageJ.

### VEGF-A quantification and neutralization

VEGF-A levels in viable-cell-normalized fibroblast-conditioned media were measured using a Human VEGF Quantikine ELISA Kit (R&D Systems, Bio-Techne, Cat. No. DVE00). For neutralization, conditioned media from hypoxic siControl- or siEDN1-treated fibroblasts were incubated for 1 h with anti-human VEGF antibody (R&D Systems, Cat. No. MAB293; 1 µg/mL) or matched mouse IgG2b control (Cat. No. MAB004; 1 µg/mL). HDMECs were seeded on growth-factor-reduced Matrigel (Corning, Cat. No. 356231) and treated with the conditioned media for 6–8 h. Total tube length and branch-point number were quantified using ImageJ.

### Collagen quantification and gel contraction assay

Collagen production was measured using the Sircol™ Collagen Assay Kit (Biocolor, Cat. No. S1000). For gel contraction assays, fibroblasts were embedded in collagen gels, which were allowed to polymerize and then detached. Gel size was measured over time to assess contractility.

### ELISA

Secreted EDN1 levels in conditioned media were quantified using an ELISA kit (R&D Systems, Cat. No. DET100) according to the manufacturer’s instructions.

### Luciferase reporter assay

Notch signaling activity was assessed using a CSL/RBPJ luciferase reporter plasmid (Addgene). Cells were co-transfected with reporter and Renilla plasmids using Lipofectamine™ 3000 (Thermo Fisher Scientific, Cat. No. L3000008). Luciferase activity was measured using the Dual-Luciferase Reporter Assay System (Promega, Cat. No. E1910) and normalized to Renilla activity.

### Interpretation of loss-of-function and dual-knockdown experiments

Functional experiments in this study were primarily performed using loss-of-function approaches, including siRNA-mediated knockdown of DLL4, NOTCH1, and EDN1, as well as combined DLL4/EDN1 knockdown. Recombinant EDN1 rescue experiments were used to assess partial functional recovery after DLL4 silencing. The study was not designed to formally distinguish additivity from synergy between DLL4 and EDN1 inhibition. Therefore, dual-knockdown results were interpreted as evidence of combined pathway dependency rather than synergistic or cooperative enhancement. Image-based quantification was performed using identical analysis settings across experimental groups. No data points were excluded unless a technical failure occurred before analysis.

### γγ-Secretase inhibition and nuclear cleaved NICD1 analysis

HDFs were pretreated with DAPT (Selleck Chemicals, Cat. No. S2215; 10 µM) or DMSO vehicle for 2 h and then maintained under normoxia or hypoxia for 24 h. Nuclear proteins were isolated using NE-PER™ Nuclear and Cytoplasmic Extraction Reagents (Thermo Fisher Scientific, Cat. No. 78833). Cleaved NICD1 was detected using a Val1744-specific anti-cleaved NOTCH1 antibody (Cell Signaling Technology, Cat. No. 4147; 1:1,000; expected molecular weight approximately 110 kDa) and normalized to Lamin B1 (Cell Signaling Technology, Cat. No. 13435; 1:1,000). In matched samples, HES1 and EDN1 mRNA levels were measured by RT-qPCR, and secreted EDN1 was quantified using a Human Endothelin-1 ELISA kit (R&D Systems, Cat. No. DET100). All experiments were performed in three independent biological replicates.

### Recombinant EDN1 rescue experiments

To evaluate whether EDN1 functionally mediated DLL4-associated fibroblast responses, HDFs were assigned to three rescue groups: hypoxia plus siControl (R1), hypoxia plus siDLL4 (R2), and hypoxia plus siDLL4 with recombinant EDN1 (R3). Following siRNA transfection, cells were exposed to hypoxia and treated with human endothelin-1 (Tocris Bioscience, Bio-Techne, Cat. No. 1160) at 50 nM for 24 h. Vehicle-treated cells were included in the R1 and R2 groups. Fibroblast proliferation was measured using the Cell Counting Kit-8 (CCK-8; Dojindo, Cat. No. CK04), whereas migration was evaluated by wound-healing assay. Profibrotic rescue was assessed by RT-qPCR analysis of COL1A1, FN1, and ACTA2, soluble collagen quantification, and collagen-gel contraction. Conditioned media from the corresponding fibroblast groups were normalized to viable donor-cell number and applied to HDMECs for endothelial tube-formation analysis. Experiments were performed using three independent biological replicates. The supplier lists Cat. No. 1160 as human/porcine endothelin-1.

#### RT-qPCR analysis of profibrotic rescue

RT-qPCR was performed according to the after mentioned protocol. The following TaqMan Gene Expression Assays were used: COL1A1, Hs00164004_m1; FN1, Hs01549976_m1; ACTA2, Hs00426835_g1; and GAPDH, Hs02786624_g1. Relative expression was calculated using the 2^^−ΔΔCt^ method with GAPDH as the endogenous control. The COL1A1 and ACTA2 assay identities are listed by Thermo Fisher as human gene-expression assays.

### Soluble collagen quantification

Fibroblast-conditioned media were collected after 24 h of recombinant EDN1 treatment and clarified by centrifugation. Soluble collagen was quantified using the Sircol Soluble Collagen Assay Kit (Biocolor, Cat. No. S1000) according to the manufacturer’s instructions. Absorbance was measured using a microplate reader, and collagen concentrations were calculated from the kit standard curve. Values were normalized to the number of viable fibroblasts present at the time of conditioned-medium collection.

### Collagen-gel contraction assay

Collagen gels were prepared using rat-tail collagen I (Corning, Cat. No. 354236). Fibroblasts from the R1–R3 groups were suspended in neutralized collagen solution, transferred to culture plates, and allowed to polymerize at 37 °C. After polymerization, the gels were gently detached from the well edges and maintained under the corresponding hypoxic treatment conditions. Gel images were acquired at baseline and after 24 h. Gel area was quantified using ImageJ, and contraction was expressed as the percentage reduction from the initial gel area. Corning identifies Cat. No. 354,236 as rat-tail collagen I suitable for gel formation.

### Public transcriptomic validation

Publicly available human keloid transcriptomic datasets GSE44270 and GSE158395 were obtained from the Gene Expression Omnibus database [20]. GSE44270 was used to compare keloid-derived fibroblasts with normal skin-derived fibroblasts, whereas GSE158395 was used to compare lesional keloid tissue with healthy skin. Processed expression matrices and sample annotations were downloaded using the GEOquery package in R. Probe or gene identifiers were converted to official gene symbols, and duplicate entries were summarized by the median expression value. The datasets were analyzed separately because of differences in sample type and profiling platform. Expression of DLL4, NOTCH1, HES1, HEY1, and EDN1 was compared between keloid and control samples using the limma package with empirical Bayes moderation. HIF1A and VEGFA were additionally evaluated as hypoxia-associated markers. Benjamini–Hochberg correction was applied for multiple comparisons, and an adjusted p value < 0.05 was considered statistically significant. A NOTCH-associated expression score was calculated from the standardized expression of DLL4, NOTCH1, HES1, and HEY1, and its association with EDN1 expression was assessed using Spearman correlation analysis. Results were visualized using boxplots and heatmaps generated with ggplot2. All analyses were performed in R.

### Statistical analysis

All experiments were performed using three independent biological replicates. A biological replicate represented an independently cultured, treated, and analyzed cell preparation. Where technical replicate wells were used, their values were averaged to obtain one value for each biological replicate and were not treated as independent observations. Data are presented as mean ± standard error of the mean (SEM). Statistical analyses were performed using GraphPad Prism 9. Comparisons between two groups were conducted using a two-tailed unpaired Student’s t-test. Comparisons involving three or more groups were performed using one-way ANOVA followed by Tukey’s multiple-comparisons test. Experiments involving two independent factors, including EDN1 silencing and VEGF neutralization, were analyzed using two-way ANOVA followed by Tukey’s multiple-comparisons test. Benjamini–Hochberg correction was applied to the public transcriptomic analyses. A p value < 0.05 was considered statistically significant. Cell-cycle distributions were analyzed separately for the G0/G1, S, and G2/M phases using one-way ANOVA followed by Tukey’s multiple-comparisons test. Only prespecified comparisons between hypoxic siControl cells and the normoxic, single-knockdown, or dual-knockdown groups were interpreted. Technical replicate values were averaged within each independent biological replicate before statistical analysis. Statistical significance was indicated as *p < 0.05, **p < 0.01, and ***p < 0.001.

## Results

### Hypoxia activates HIF1A/VEGFA, DLL4/NOTCH, and endothelin signaling in fibroblasts

**Fig. 1:**
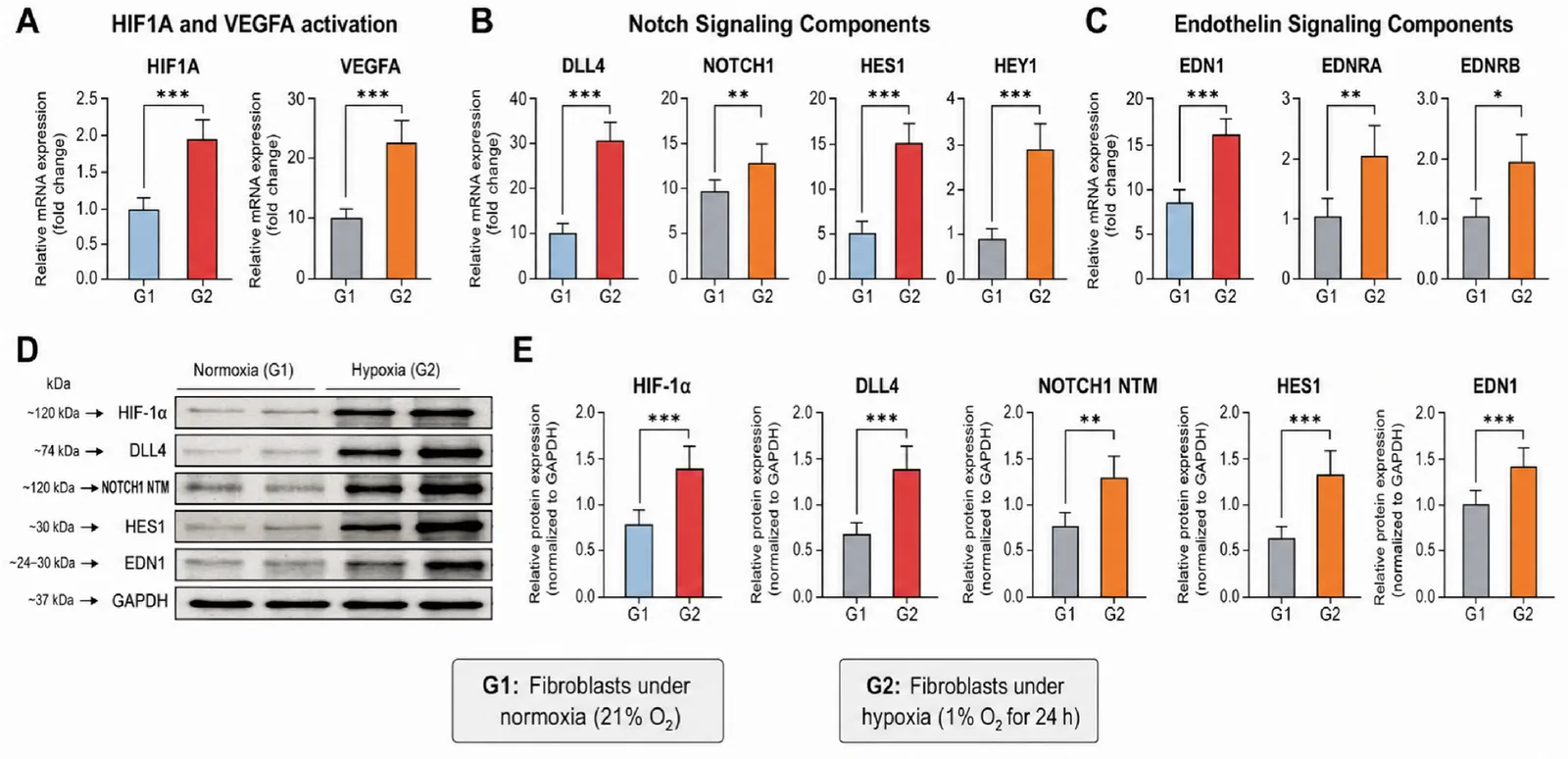
Hypoxia activates HIF1A/VEGFA, DLL4-associated NOTCH, and endothelin signaling in fibroblasts. **A **RT-qPCR analysis of HIF1A and VEGFA in fibroblasts cultured under normoxic conditions or hypoxia at 1% O₂ for 24 h. **B **RT-qPCR analysis of DLL4, NOTCH1, HES1, and HEY1. **C **RT-qPCR analysis of EDN1, EDNRA, and EDNRB. **D **Representative Western blots showing HIF-1α (~120 kDa), DLL4 (~74 kDa), NOTCH1 NTM (~120 kDa), HES1 (~30 kDa), EDN1 (~24–30 kDa), and GAPDH (~37 kDa). The NOTCH1 antibody used in this experiment was not cleavage-specific for activated NICD1. **E **Densitometric quantification of protein expression normalized to GAPDH. Data are presented as mean ± SEM (*n* = 3). *p < 0.05, **p < 0.01, ***p < 0.001

To assess hypoxia-responsive signaling in the in vitro fibroblast model, normal human dermal fibroblasts were cultured under normoxic (21% O₂) or hypoxic (1% O₂, 24 h) conditions and analyzed by RT-qPCR and Western blotting (Fig. 1). RT-qPCR revealed that hypoxia significantly increased expression of HIF1A and VEGFA (both *p* < 0.001; Fig. 1A), confirming effective hypoxic stimulation. Hypoxia increased DLL4, the experimentally assessed receptor NOTCH1, and the canonical NOTCH targets HES1 and HEY1 (*p* < 0.001, *p* < 0.01, and both *p* < 0.001, respectively; Fig. 1B), supporting activation of DLL4-associated NOTCH signaling. In parallel, the endothelin pathway was induced, as shown by increased EDN1 (*p* < 0.001), EDNRA (*p* < 0.01), and EDNRB (*p* < 0.05) expression (Fig. 1C). Consistent with these findings, Western blot analysis further validated these findings at the protein level (Fig. 1D-E and Figure S1). Hypoxia markedly increased HIF-1α protein expression, confirming stabilization of the hypoxia-inducible factor. In parallel, DLL4, the approximately 120-kDa NOTCH1 NTM form, and the downstream effector HES1 were significantly elevated under hypoxic conditions. Moreover, EDN1 protein levels were also strongly increased, supporting activation of the endothelin signaling axis. Densitometric quantification revealed significant increases in HIF-1α (*p* < 0.001), DLL4 (*p* < 0.001), NOTCH1 NTM (*p* < 0.01), HES1 (*p* < 0.001), and EDN1 (*p* < 0.001) relative to normoxic controls (Fig. 1E). Because the NOTCH1 antibody used in this experiment was not cleavage-specific, the approximately 120-kDa signal was interpreted as NOTCH1 NTM abundance rather than activated NICD1. Together, these results indicate that hypoxia induces HIF1A/VEGFA signaling, increases DLL4 and NOTCH1 NTM abundance, activates canonical NOTCH-associated transcriptional targets, and induces the EDN1 endothelin pathway.

### TGF-β1 induces a profibrotic fibroblast phenotype and upregulates DLL4 and EDN1 expression

**Fig. 2:**
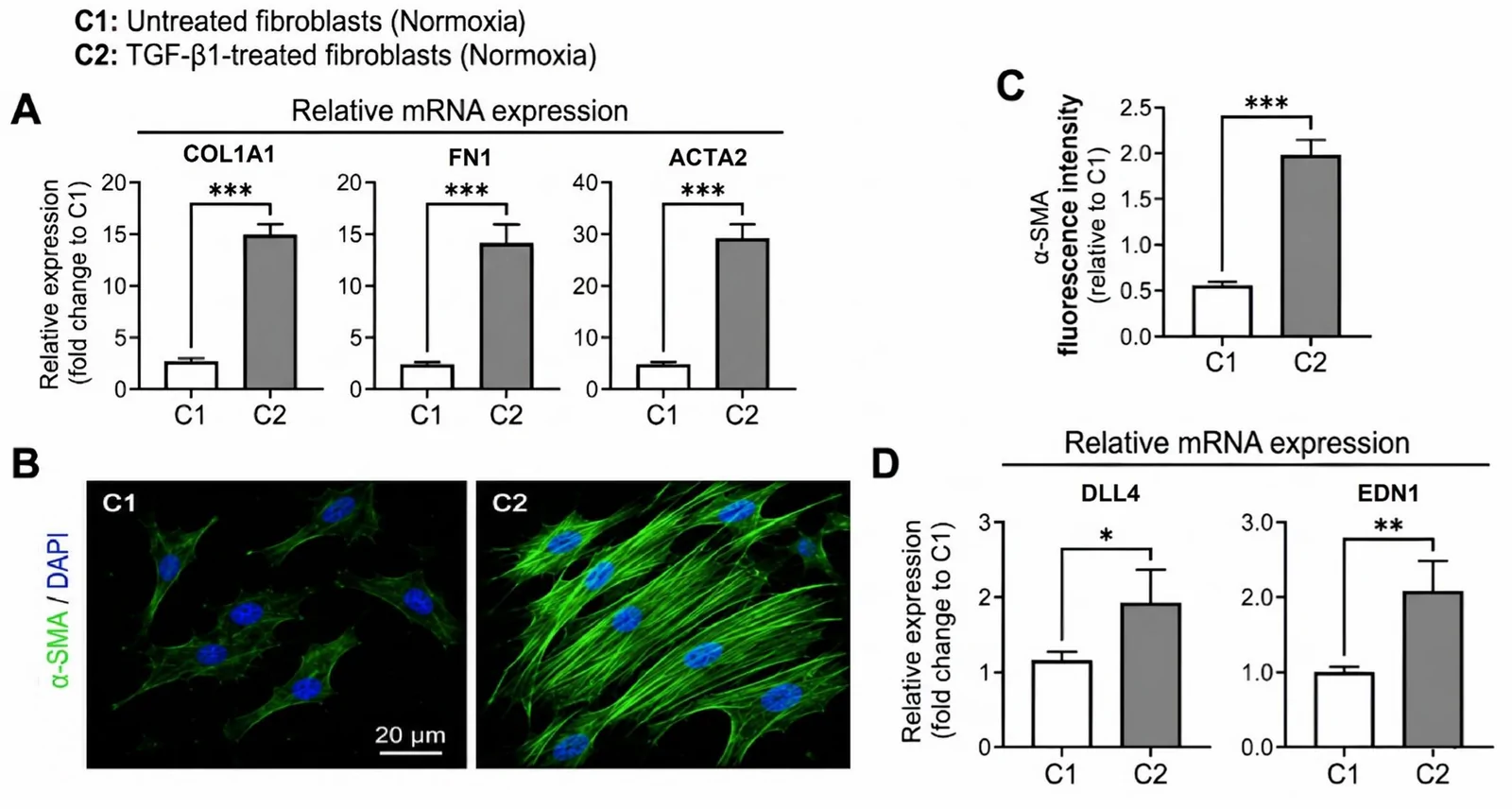
TGF-β1 induces a profibrotic fibroblast phenotype and increases DLL4 and EDN1 expression. **A **RT-qPCR analysis of COL1A1, FN1, and ACTA2 expression. **B **Higher-magnification representative immunofluorescence images showing α-SMA-positive stress fibers (green) and DAPI-stained nuclei (blue). Scale bar: 20 µm. **C **Quantification of background-corrected α-SMA fluorescence normalized to DAPI-positive cell number. Five randomly selected fields were analyzed per biological replicate. **D **RT-qPCR analysis of DLL4 and EDN1 expression. Data are presented as mean ± SEM from three independent biological replicates. *p < 0.05, **p < 0.01, ***p < 0.001

To establish an in vitro fibrotic model, fibroblasts were treated with TGF-β1 and analyzed for profibrotic markers (Fig. 2). TGF-β1 significantly increased the mRNA expression of COL1A1, FN1, and ACTA2 compared with untreated controls (all *p* < 0.001; Fig. 2A). Higher-magnification immunofluorescence images showed increased α-SMA-positive stress-fiber formation in TGF-β1-treated fibroblasts. ImageJ-based quantification of background-corrected α-SMA fluorescence normalized to DAPI-positive cell number confirmed a significant increase relative to untreated controls (*p* < 0.001; Fig. 2B–C). In addition, TGF-β1 induced a modest but significant upregulation of DLL4 (*p* < 0.05) and EDN1 (*p* < 0.01) expression (Fig. 2D), suggesting that fibrotic activation is associated with early induction of the DLL4/NOTCH and endothelin signaling axes. These results indicate that TGF-β1 drives a profibrotic/myofibroblast-like phenotype in fibroblasts and is accompanied by activation of pathways implicated in angiogenesis and fibrotic signaling.

### Efficient siRNA-mediated target suppression in fibroblasts

**Fig. 3:**
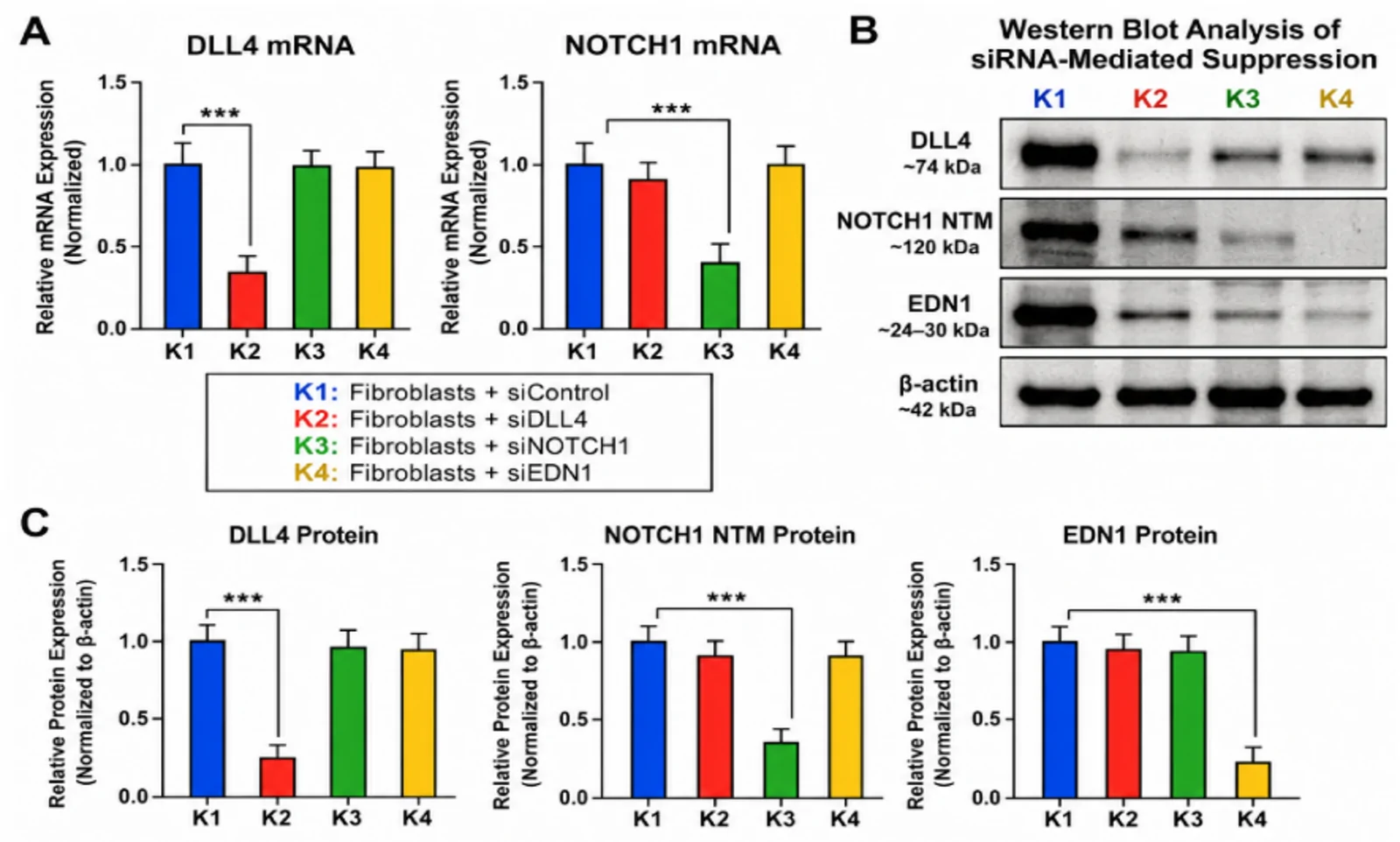
Efficient siRNA-mediated target suppression in fibroblasts. **A **RT-qPCR validation of DLL4 and NOTCH1 knockdown. **B **Representative Western blots showing DLL4 (~74 kDa), the NOTCH1 transmembrane/intracellular form (NOTCH1 NTM; ~120 kDa), EDN1 (~24–30 kDa), and β-actin (~42 kDa) after the indicated siRNA treatments. The NOTCH1 antibody used in this experiment was not cleavage-specific for activated NICD1. **C **Densitometric quantification of the indicated proteins normalized to β-actin. Data are presented as mean ± SEM. ***p < 0.001

To validate the efficiency of gene silencing, fibroblasts were transfected with siRNAs targeting DLL4 (K2), NOTCH1 (K3), or EDN1 (K4) and compared with siControl-transfected cells (K1) (Fig. 3). RT-qPCR analysis demonstrated that DLL4 mRNA expression was significantly reduced following siDLL4 transfection (K2 vs. K1, *p* < 0.001), confirming effective DLL4 suppression (Fig. 3A). Similarly, NOTCH1 mRNA levels were markedly decreased in siNOTCH1-treated cells (K3 vs. K1, *p* < 0.001), confirming efficient NOTCH1 knockdown (Fig. 3A). Consistent with the target-validation data, Western blot analysis confirmed reduced protein expression of the corresponding targets (Fig. 3B). Specifically, DLL4 protein levels were decreased in K2, the approximately 120-kDa NOTCH1 NTM form was reduced in K3, and EDN1 expression was diminished in K4, while β-actin remained unchanged across all groups, indicating comparable protein loading (Fig. 3B and Figure S1). Densitometric quantification further demonstrated significant reductions in DLL4 protein (K2 vs. K1, *p* < 0.001), NOTCH1 NTM protein (K3 vs. K1, *p* < 0.001), and EDN1 protein (K4 vs. K1, *p* < 0.001) relative to control cells (Fig. 3C). Collectively, these results confirm efficient siRNA-mediated suppression of DLL4, NOTCH1, and EDN1 at the mRNA and/or protein levels. EDN1 regulation by DLL4/NOTCH knockdown and HES1 feedback after EDN1 silencing were evaluated separately under hypoxic functional conditions in next part.

### DLL4 and EDN1 are required to maintain hypoxia-associated fibroblast proliferation

**Fig. 4:**
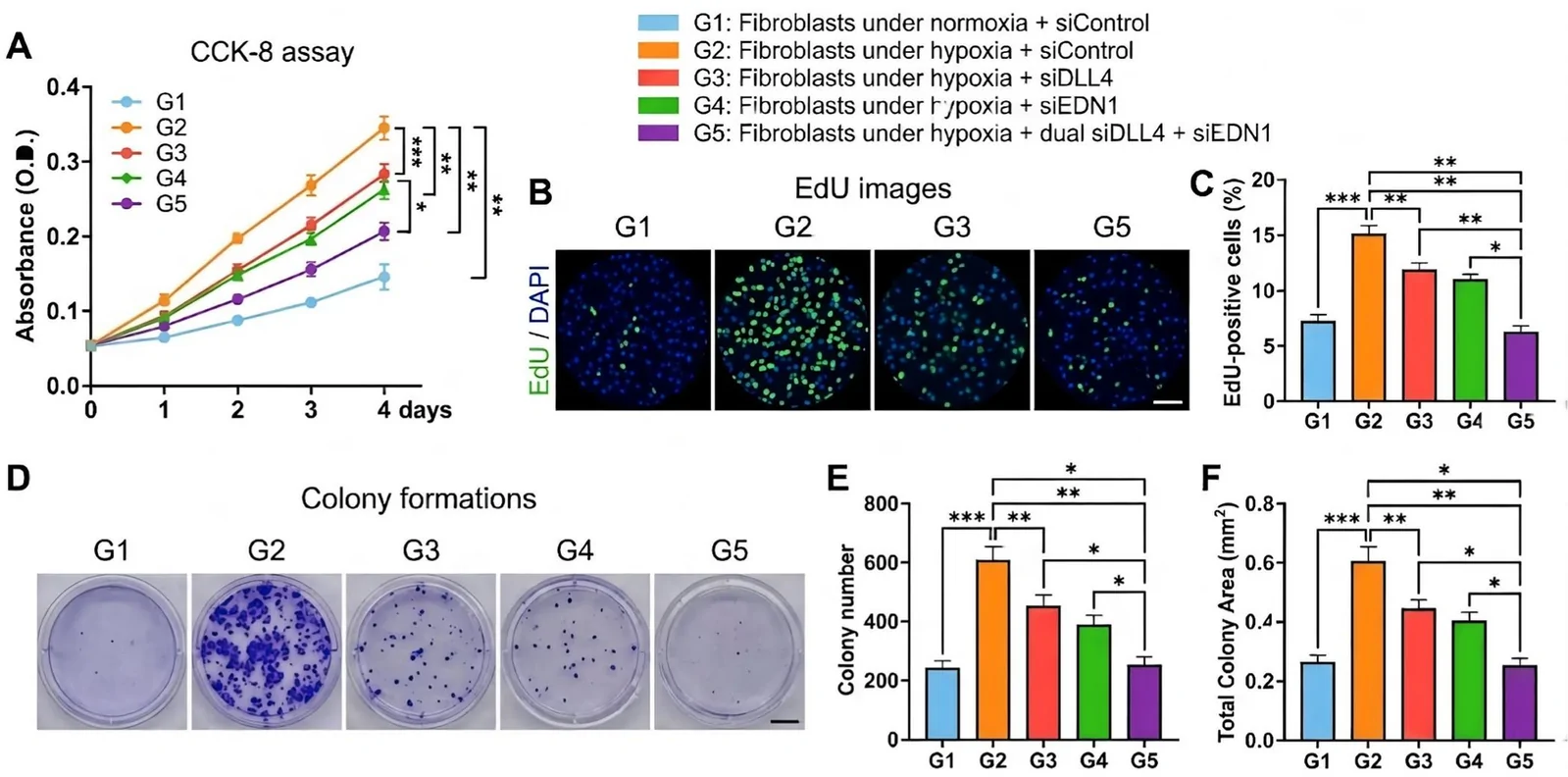
DLL4 and EDN1 are required to maintain hypoxia-associated fibroblast proliferation. **A **CCK-8 assay showing fibroblast proliferation over time. **B **Representative EdU staining images (green: EdU; blue: DAPI). **C **Quantification of EdU-positive cells. **D **Representative colony formation images. **E** Quantification of colony number. **F **Quantification of total colony area. Mean ± SEM. ***p<0.001, **p<0.01, p*<0.05

We next examined whether DLL4 and EDN1 are required to maintain hypoxia-associated fibroblast proliferation (Fig. 4). In the CCK-8 assay, hypoxia significantly increased fibroblast growth relative to normoxic controls (G2 vs. G1, *p* < 0.001; Fig. 4A). Knockdown of DLL4 or EDN1 each significantly reduced proliferation under hypoxia, whereas combined DLL4 and EDN1 knockdown produced the strongest inhibitory effect relative to hypoxic control cells (G5 vs. G2, *p* < 0.001). Dual knockdown also significantly suppressed proliferation compared with either single knockdown group (G5 vs. G3 and G5 vs. G4, *p* < 0.05 to *p* < 0.01, as indicated). EdU incorporation assays showed a similar pattern. Hypoxia increased the proportion of EdU-positive fibroblasts compared with normoxia (G2 vs. G1, *p* < 0.001; Fig. 4B-C). DLL4 or EDN1 knockdown significantly reduced DNA synthesis under hypoxia (G3 vs. G2 and G4 vs. G2, both *p* < 0.01), while dual knockdown further decreased EdU incorporation relative to hypoxic controls (G5 vs. G2, *p* < 0.01) and to each single knockdown group (G5 vs. G3, *p* < 0.01; G5 vs. G4, *p* < 0.05). Colony formation assays further confirmed these findings. Hypoxia markedly increased both colony number and total colony area compared with normoxia (G2 vs. G1, *p* < 0.001; Fig. 4D–F). Silencing DLL4 or EDN1 reduced colony-forming capacity relative to hypoxic control cells (G3 vs. G2 and G4 vs. G2, generally *p* < 0.01), whereas dual knockdown caused the most pronounced decrease (G5 vs. G2, *p* < 0.001). In addition, dual knockdown significantly reduced colony number and colony area compared with either single knockdown condition (G5 vs. G3 and G5 vs. G4, *p* < 0.05). Together, these results indicate that DLL4 and EDN1 are required to maintain hypoxia-associated fibroblast proliferation, DNA synthesis, and colony-forming capacity, with combined knockdown producing the strongest reduction.

### DLL4 and EDN1 are required for hypoxia-associated survival and cell-cycle progression

**Fig. 5:**
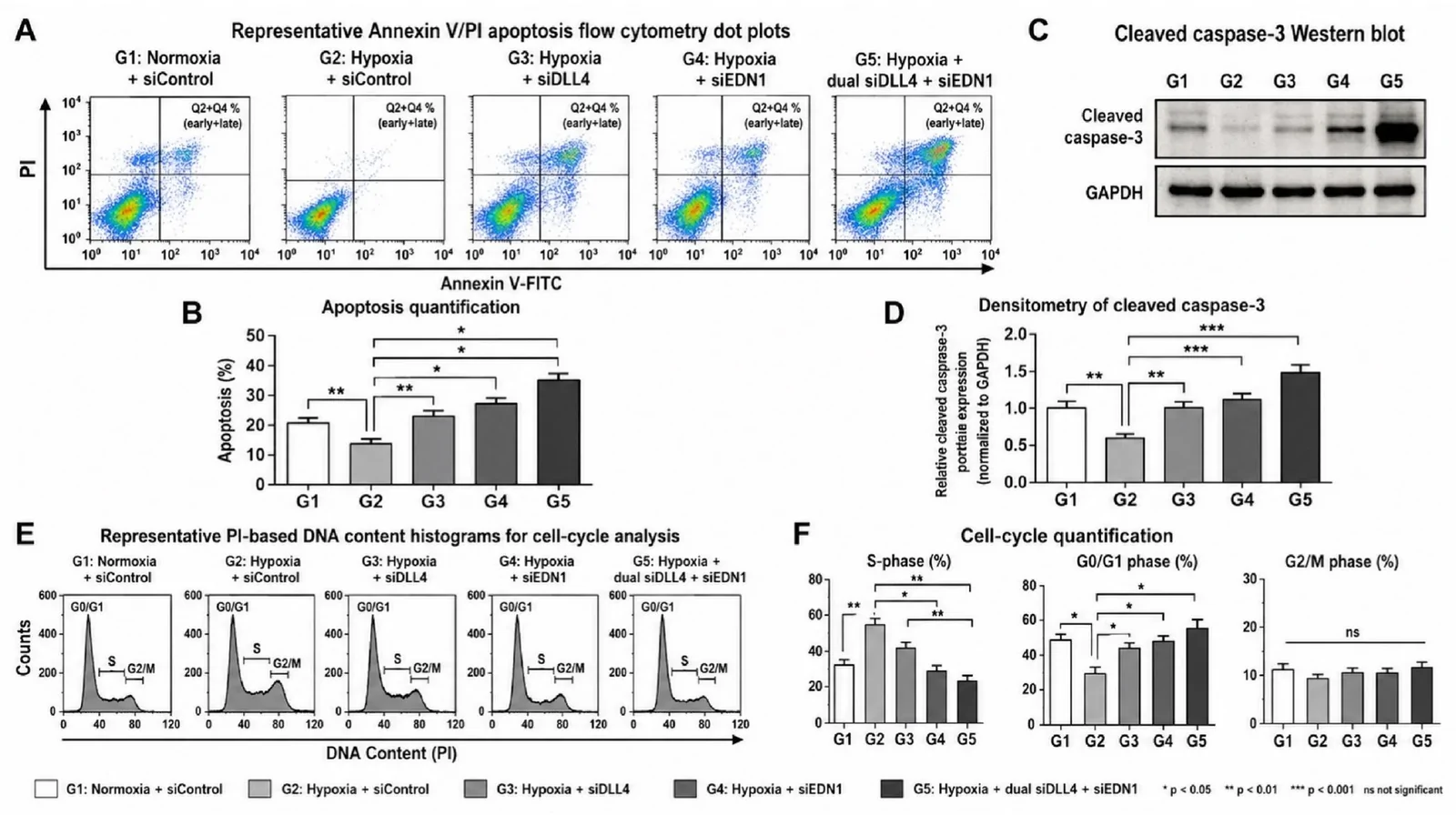
DLL4 and EDN1 silencing increased apoptosis and altered cell-cycle progression in hypoxic human dermal fibroblasts. **A **Representative Annexin V-FITC/PI flow-cytometry plots showing viable, early apoptotic, late apoptotic, and necrotic fibroblast populations. **B **Quantification of total apoptotic cells, calculated as the sum of early and late apoptotic populations. **C **Representative Western blot images of cleaved caspase-3 and GAPDH. **D **Densitometric quantification of cleaved caspase-3 expression normalized to GAPDH. **E **Representative flow-cytometry histograms showing cell-cycle distribution. **F** Quantification of the proportions of cells in the G0/G1, S, and G2/M phases. Groups were defined as normoxia plus siControl (G1), hypoxia plus siControl (G2), hypoxia plus siDLL4 (G3), hypoxia plus siEDN1 (G4), and hypoxia plus combined siDLL4 and siEDN1 (G5). Data are presented as mean ± SEM from three independent biological replicates. Statistical comparisons were performed using one-way ANOVA followed by Tukey’s multiple-comparisons test. Cell-cycle phases were analyzed separately. *p < 0.05, **p < 0.01, and ***p < 0.001; ns, not significant

We next assessed whether the hypoxia-induced proliferative phenotype was associated with altered apoptosis and cell-cycle progression (Fig. 5). Annexin V/PI flow cytometry showed that hypoxia significantly reduced the proportion of apoptotic fibroblasts compared with normoxia (G2 vs. G1, *p* < 0.01; Fig. 5A-B). Knockdown of DLL4 significantly increased apoptosis relative to hypoxic siControl cells (G3 vs. G2, *p* < 0.01), while EDN1 knockdown also accelerated apoptosis (G4 vs. G2, *p* < 0.05). Combined DLL4 and EDN1 knockdown (G5) resulted in the highest level of apoptosis compared with hypoxic control cells (G5 vs. G2, *p* < 0.05; Fig. 5B). Western blot analysis of cleaved caspase-3 supported these findings, with expressions normalized to GAPDH (Fig. 5C-D). Cleaved caspase-3 expression was reduced under hypoxia compared with normoxia (G2 vs. G1, *p* < 0.01). Conversely, expression significantly increased following DLL4 knockdown (G3 vs. G2, *p* < 0.01) or EDN1 knockdown (G4 vs. G2, *p* < 0.001). The highest cleaved caspase-3 levels were observed in the dual knockdown group, which was strongly elevated compared with hypoxic control cells (G5 vs. G2, *p* < 0.001; Fig. 5D). Cell-cycle analysis showed that hypoxia significantly increased the S-phase fraction compared with normoxic controls (G2 vs. G1, *p* < 0.01; Fig. 5E-F). Silencing either target significantly reduced the S-phase fraction under hypoxia (G3 vs. G2, *p* < 0.05; G4 vs. G2, *p* < 0.01). Dual DLL4/EDN1 knockdown produced the lowest S-phase fraction relative to hypoxic siControl cells (G5 vs. G2, *p* < 0.01). Concurrently, hypoxia reduced the G0/G1-phase fraction compared with normoxia (G2 vs. G1, *p* < 0.05). Knockdown of DLL4, EDN1, or both targets combined significantly restored the G0/G1 fraction relative to hypoxic siControl cells (all comparisons vs. G2, *p* < 0.05; Fig. 5F). No significant differences were observed in the G2/M phase across any groups. Together, these findings indicate that DLL4 and EDN1 are required to sustain hypoxia-associated apoptosis suppression and cell-cycle progression.

### DLL4 and EDN1 silencing attenuated hypoxia-associated fibroblast motility and invasion

**Fig. 6:**
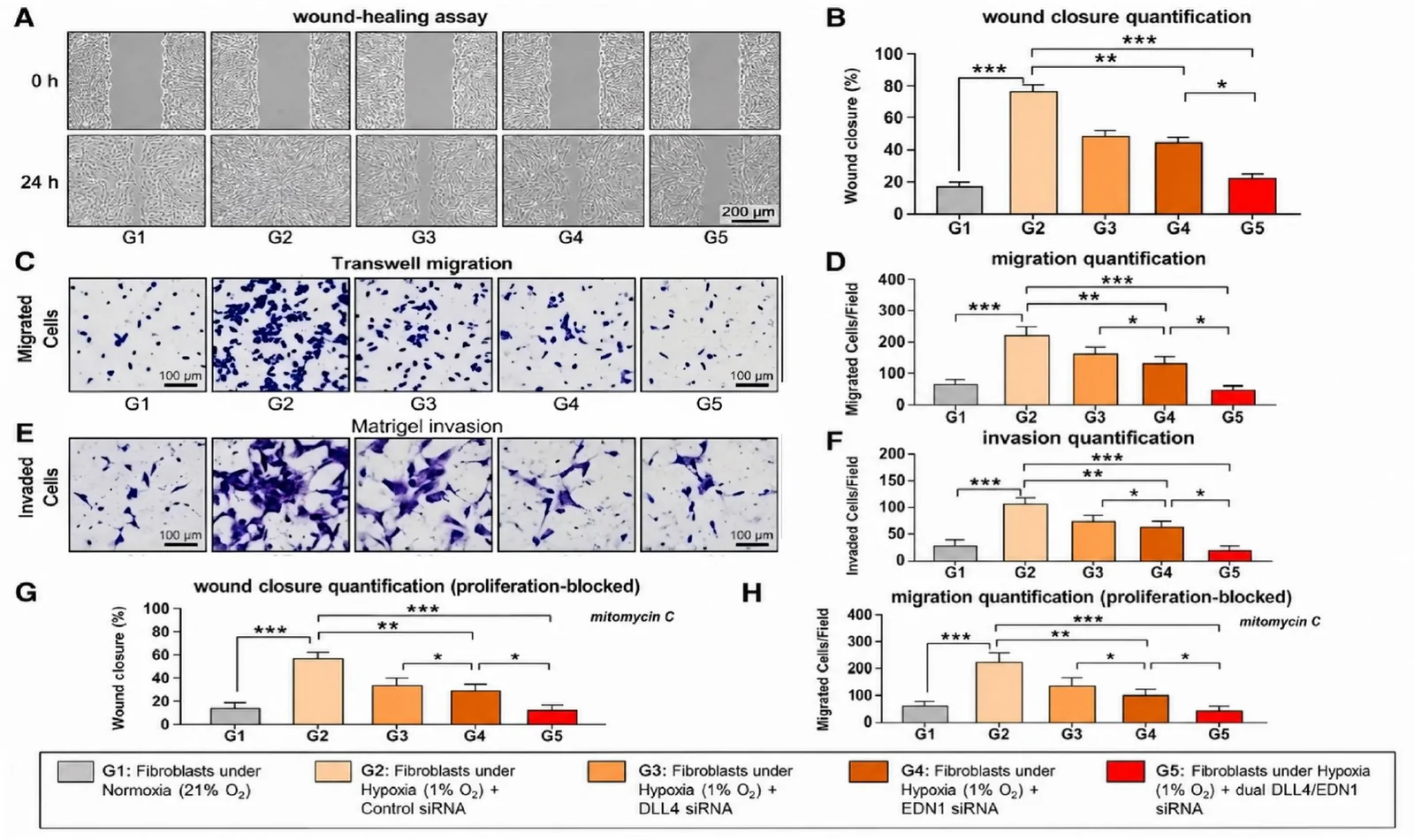
DLL4 and EDN1 are required to maintain hypoxia-associated fibroblast migration and invasion. **A **Representative wound-healing images at 0 h and 24 h. **B **Quantification of wound closure (%). **C **Representative Transwell migration images. **D **Quantification of migrated cells. **E **Representative Matrigel invasion images. **F **Quantification of invaded cells. Mean ± SEM. ***p<0.001, **p<0.01, p*<0.05

To determine whether DLL4 and EDN1 contributed to hypoxia-associated fibroblast motility, wound-healing, Transwell migration, and Matrigel-invasion assays were performed (Fig. 6). Hypoxia significantly increased wound closure compared with normoxia (G2 vs. G1, *p* < 0.001; Fig. 6A-B). DLL4 or EDN1 silencing significantly reduced wound closure relative to hypoxic siControl cells (both groups vs. G2, *p* < 0.01), whereas combined DLL4/EDN1 knockdown produced the greatest reduction (G5 vs. G2, *p* < 0.001; G5 vs. G4, *p* < 0.05). Transwell analysis showed that hypoxia significantly increased fibroblast migration compared with normoxia (G2 vs. G1, *p* < 0.001; Fig. 6C-D). Knockdown of DLL4 (*p* < 0.01) or EDN1 (*p* < 0.001) markedly reduced migration compared to hypoxic controls. Combined knockdown resulted in the lowest migrated-cell number (G5 vs. G2, *p* < 0.001), which was significantly decreased compared to either single knockdown group (G5 vs. G3 and G5 vs. G4, both *p* < 0.05). Matrigel-invasion assays showed a similar pattern (Fig. 6E-F); hypoxia increased fibroblast invasion relative to normoxia (*p* < 0.001), whereas DLL4 (*p* < 0.01) or EDN1 (*p* < 0.05) silencing significantly attenuated invasion. Dual DLL4/EDN1 knockdown produced the strongest reduction in invaded cells (G5 vs. G2, *p* < 0.001; *p* < 0.05 vs. single-knockdown groups). Because DLL4 and EDN1 silencing also affected fibroblast proliferation, wound-healing and Transwell migration assays were repeated under proliferation-blocked conditions utilizing mitomycin C (Fig. 6G-H). Following mitomycin C treatment, hypoxia continued to increase wound closure and migration compared with normoxia (both *p* < 0.001). Under these proliferation-blocked conditions, individual silencing of DLL4 or EDN1 still significantly attenuated wound closure (both *p* < 0.01; Fig. 6G) and migration (G3 vs. G2, *p* < 0.01; G4 vs. G2, *p* < 0.001; Fig. 6H). Combined knockdown consistently yielded the greatest reduction overall (*p* < 0.001 vs. G2; *p* < 0.05 vs. the single-knockdown cohorts). These findings confirm that DLL4- and EDN1-associated changes in fibroblast motility were direct effects and not driven solely by differences in cell proliferation.

### DLL4 and EDN1 are required for hypoxia-associated fibrotic remodeling and contractile activity

**Fig. 7:**
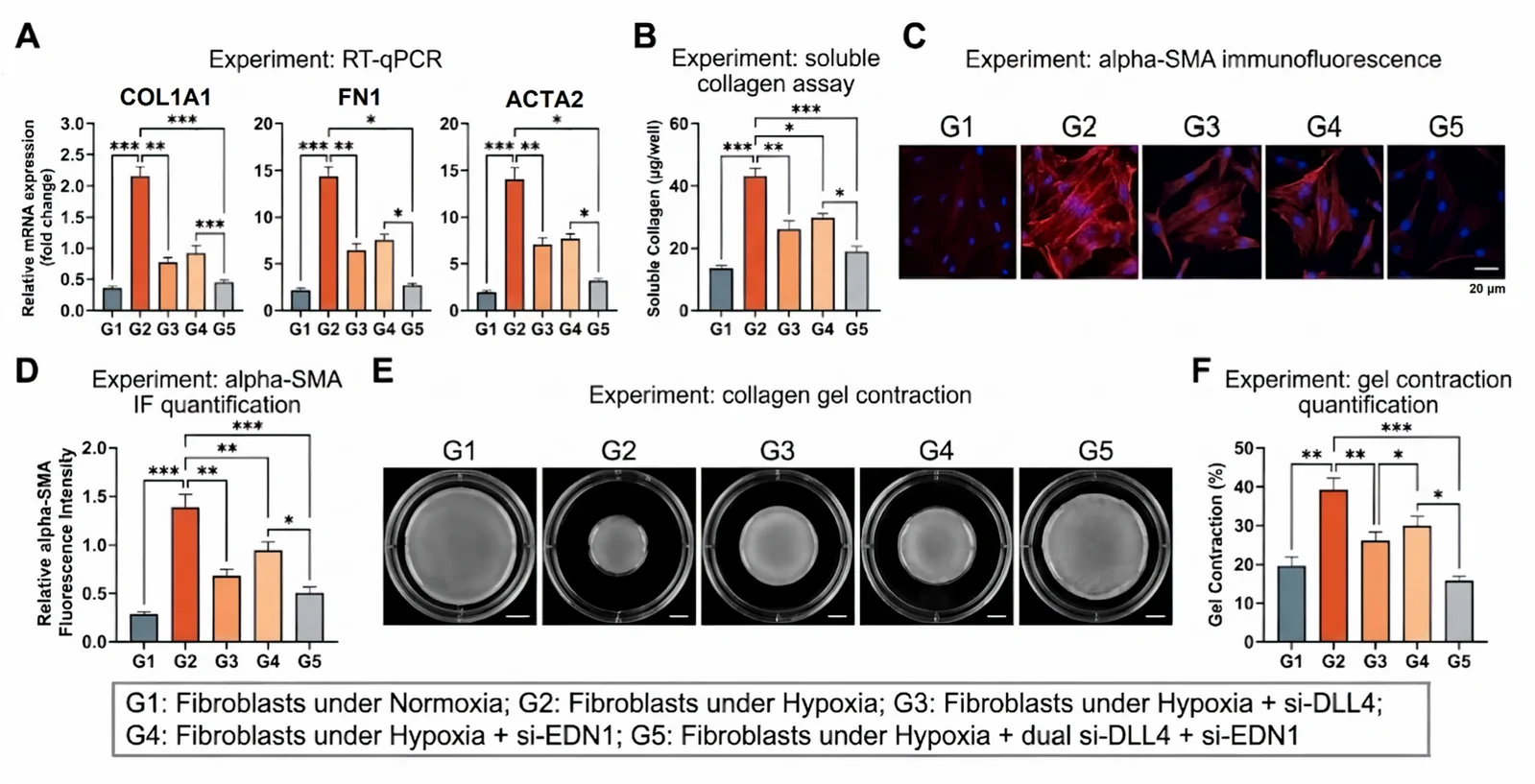
DLL4 and EDN1 are required for hypoxia-associated fibrotic remodeling and contractile activity. **A **RT-qPCR analysis of fibrotic genes (COL1A1, FN1, ACTA2). **B **Soluble collagen assay quantification. **C **Representative α-SMA immunofluorescence images. **D **Quantification of α-SMA fluorescence intensity. **E **Collagen gel contraction assay images. **F **Quantification of gel contraction (%). Mean ± SEM. ***p<0.001, **p<0.01, p*<0.05

Given that keloid progression is characterized by excessive extracellular matrix deposition and fibroblast activation, we next assessed fibrotic remodeling under hypoxic conditions (Fig. 7). RT-qPCR analysis showed that hypoxia significantly increased COL1A1, FN1, and ACTA2 expression compared with normoxia (G2 vs. G1, *p* < 0.001; Fig. 7A). Knockdown of DLL4 significantly reduced the expression of all three fibrotic markers relative to the hypoxic control (G3 vs. G2, *p* < 0.01), whereas EDN1 silencing alone (G4) did not produce a statistically significant decrease compared to G2. Combined dual knockdown of DLL4 and EDN1 produced a highly significant suppression of COL1A1 relative to the hypoxic control (G5 vs. G2, *p* < 0.001) and a significant suppression of FN1 and ACTA2 (G5 vs. G2, *p* < 0.05). Furthermore, dual knockdown significantly reduced the expression of all three markers compared to the single EDN1 knockdown group (G5 vs. G4, *p* < 0.001 for COL1A1; *p* < 0.05 for FN1 and ACTA2). Consistent with these findings, soluble collagen assays demonstrated that hypoxia significantly enhanced collagen production (G2 vs. G1, *p* < 0.001; Fig. 7B). Silencing DLL4 significantly decreased collagen levels compared to the hypoxic control (G3 vs. G2, *p* < 0.01), whereas EDN1 knockdown alone did not result in a significant change. Combined DLL4 and EDN1 inhibition resulted in the greatest decrease in collagen production relative to the hypoxic control (G5 vs. G2, *p* < 0.001) and was significantly lower than the single EDN1 knockdown group (G5 vs. G4, *p* < 0.05). alpha-SMA immunofluorescence further confirmed fibroblast activation, with hypoxia markedly increasing alpha-SMA intensity and stress fiber organization (G2 vs. G1, *p* < 0.001; Fig. 7C-D). DLL4 knockdown significantly reduced alpha-SMA expression relative to the hypoxic control (G3 vs. G2, *p* < 0.01). Dual knockdown produced the most pronounced suppression compared to the hypoxic control (G5 vs. G2, *p* < 0.001) and further reduced alpha-SMA expression compared to the single EDN1 knockdown group (G5 vs. G4, *p* < 0.05). Finally, collagen gel contraction assays showed that hypoxia significantly increased fibroblast contractility (G2 vs. G1, *p* < 0.01; Fig. 7E-F). Knockdown of DLL4 significantly reduced contractility (G3 vs. G2, *p* < 0.01), whereas EDN1 knockdown alone did not show a significant difference against G2. Dual inhibition exerted the strongest suppressive effect relative to the hypoxic control (G5 vs. G2, *p* < 0.001) and significantly lowered contractility compared to the single EDN1 knockdown group (G5 vs. G4, *p* < 0.05). Collectively, these results indicate that DLL4 and EDN1 are required to maintain hypoxia-associated extracellular matrix production, myofibroblast activation, and contractile function, with the effects driven primarily by DLL4 or dual target inhibition.

### DLL4/EDN1 perturbation and VEGF neutralization altered endothelial responses to hypoxic fibroblast-conditioned medium

**Fig. 8:**
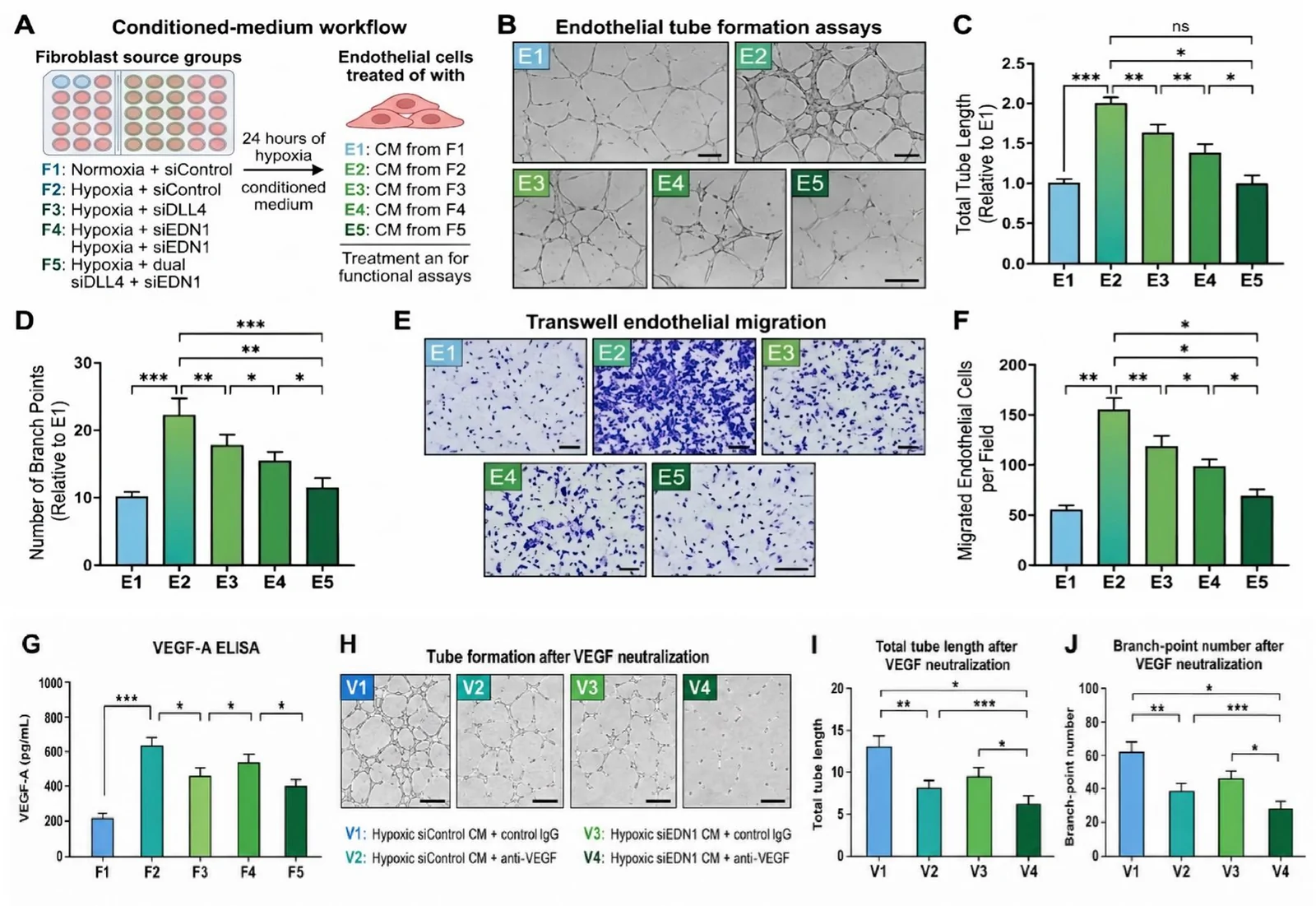
DLL4/EDN1 perturbation and VEGF neutralization alter endothelial responses to hypoxic fibroblast-conditioned medium. **A **Schematic of conditioned-medium collection and HDMEC treatment. Fibroblast groups were defined as F1, normoxia + siControl; F2, hypoxia + siControl; F3, hypoxia + siDLL4; F4, hypoxia + siEDN1; and F5, hypoxia + combined siDLL4/siEDN1. Corresponding endothelial groups were designated E1–E5. **B **Representative HDMEC tube-formation images following treatment with the indicated conditioned media. **C **Quantification of total tube length. **D** Quantification of branch-point number. **E **Representative Transwell migration images. **F **Quantification of migrated endothelial cells. **G **ELISA quantification of VEGF-A in fibroblast-conditioned media. **H **Representative HDMEC tube-formation images following VEGF neutralization. Neutralization groups were defined as V1, hypoxic siControl conditioned medium + control IgG; V2, hypoxic siControl conditioned medium + anti-VEGF; V3, hypoxic siEDN1 conditioned medium + control IgG; and V4, hypoxic siEDN1 conditioned medium + anti-VEGF. **I **Quantification of total tube length following VEGF neutralization. **J **Quantification of branch-point number following VEGF neutralization. Data are presented as mean ± SEM from three independent biological replicates. *p < 0.05, **p < 0.01, ***p < 0.001; ns, not significant

To examine how donor-fibroblast signaling influenced endothelial responses, conditioned media from fibroblast groups F1-F5 were applied to HDMECs (Fig. 8A). Conditioned medium from hypoxic siControl fibroblasts significantly increased endothelial total tube length and branch-point number compared with normoxic conditioned medium (E2 vs. E1, both *p* < 0.001; Fig. 8B-D). Silencing either DLL4 or EDN1 in donor fibroblasts significantly attenuated both total tube length and branch-point number compared to the hypoxic control group (E3 vs. E2 and E4 vs. E2, both *p* < 0.01; Fig. 8C-D). For total tube length, the dual knockdown group showed a significant decrease compared to the hypoxic control group (E5 vs. E2, *p* < 0.05) and compared to the single EDN1 knockdown group (E5 vs. E4, *p* < 0.05), reducing tube length to a level that was no longer significantly different from the normoxic control group (E5 vs. E1, ns; Fig. 8C). For branch points, combined DLL4/EDN1 knockdown produced the greatest overall reduction relative to hypoxic siControl-conditioned medium (E5 vs. E2, *p* < 0.001; Fig. 8D). Endothelial migration showed a similar pattern. Hypoxic fibroblast-conditioned medium significantly increased HDMEC migration compared with normoxic conditioned medium (E2 vs. E1, *p* < 0.01; Fig. 8E-F). Conditioned media from DLL4-silenced fibroblasts (E3 vs. E2, *p* < 0.01) or EDN1-silenced fibroblasts (E4 vs. E2, *p* < 0.05) significantly reduced endothelial migration. Dual DLL4/EDN1 knockdown produced a significant attenuation relative to the hypoxic control (E5 vs. E2, *p* < 0.05) and further decreased migration compared to the single EDN1 knockdown group (E5 vs. E4, *p* < 0.05; Fig. 8F). VEGF-A ELISA showed that hypoxia significantly increased VEGF-A secretion compared with normoxia (F2 vs. F1, *p* < 0.001; Fig. 8G). VEGF-A levels were significantly reduced following DLL4, EDN1, or combined DLL4/EDN1 knockdown relative to hypoxic siControl fibroblasts (all comparisons vs. F2, *p* < 0.05). The functional contribution of VEGF was further evaluated using conditioned media from hypoxic siControl- and siEDN1-transfected fibroblasts in the presence of a VEGF-neutralizing antibody or matched IgG control. VEGF neutralization significantly reduced endothelial total tube length and branch-point number in HDMECs treated with hypoxic siControl-conditioned medium (V2 vs. V1, *p* < 0.01; Fig. 8H-J). Conditioned medium from EDN1-silenced fibroblasts also significantly reduced both parameters compared to the control group (V3 vs. V1, *p* < 0.01). Combined EDN1 silencing and VEGF neutralization produced an additional decrease compared to the control group (V4 vs. V1, *p* < 0.05) and resulted in a highly significant reduction compared to EDN1 silencing alone with control IgG (V4 vs. V3, *p* < 0.001). Two-way ANOVA showed significant main effects of EDN1 silencing and VEGF neutralization (both *p* < 0.01), without a significant interaction between the two factors (*p* > 0.05). Collectively, these findings show that hypoxic fibroblast-conditioned medium promoted endothelial migration and tube formation through a multifactorial secretome. Perturbation of fibroblast DLL4/NOTCH or EDN1 signaling altered the endothelial-supportive activity of the conditioned medium, while VEGF neutralization provided independent evidence for a contribution from VEGF.

### DLL4/NOTCH and EDN1 signaling exhibit functional crosstalk in fibroblasts

**Fig. 9:**
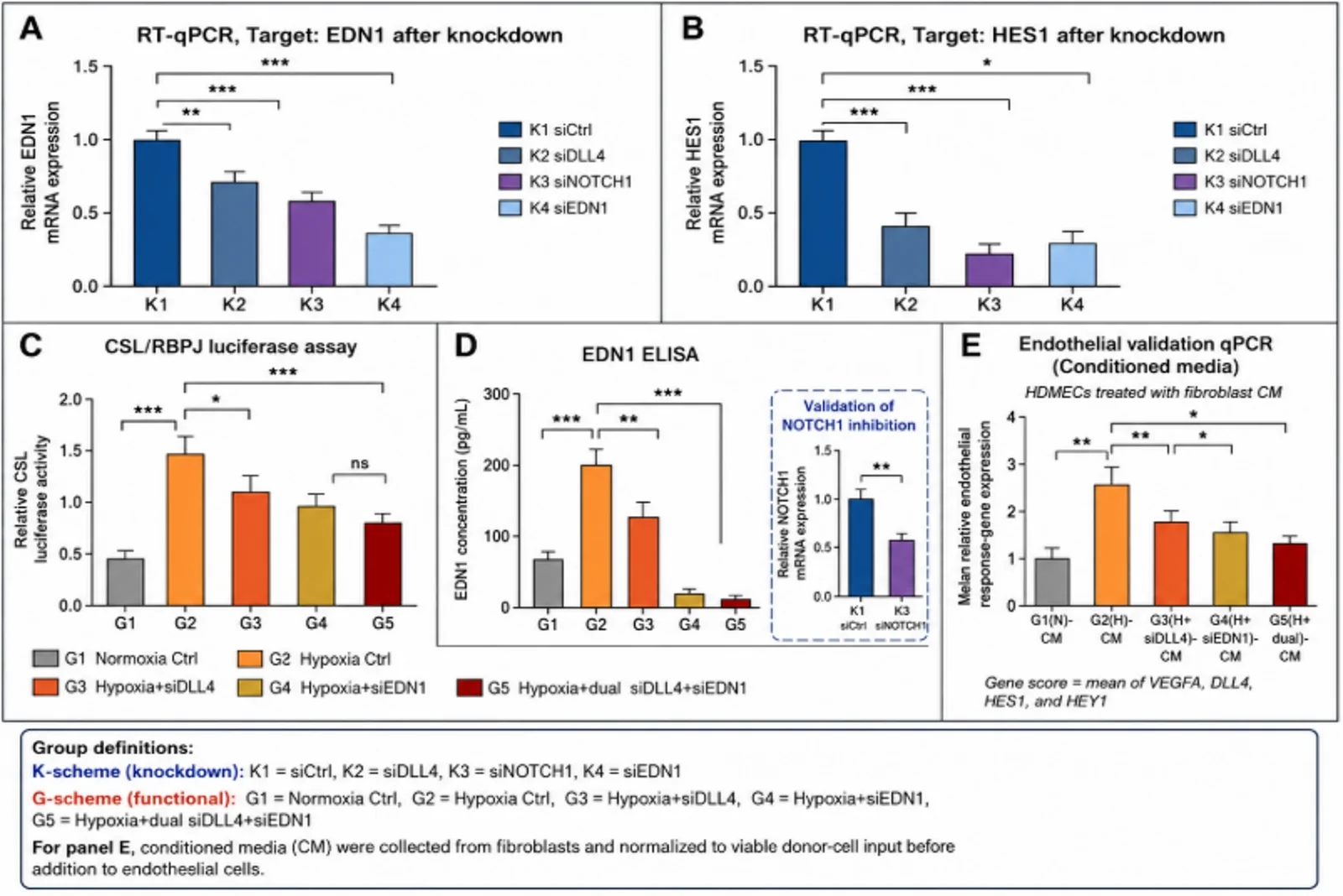
DLL4/NOTCH and EDN1 signaling exhibit asymmetric bidirectional crosstalk in fibroblasts. **A **RT-qPCR analysis of EDN1 mRNA expression after siRNA-mediated knockdown of DLL4, NOTCH1, or EDN1 under hypoxic crosstalk conditions. **B **RT-qPCR analysis of HES1 mRNA expression after the indicated knockdowns, showing strong reduction after DLL4 or NOTCH1 silencing and modest reduction after EDN1 silencing. **C **CSL/RBPJ luciferase reporter assay showing hypoxia-induced NOTCH transcriptional activity and its attenuation after DLL4, EDN1, or dual DLL4/EDN1 knockdown. **D ** ELISA quantification of secreted EDN1 in conditioned media from normoxic control, hypoxic control, hypoxic siDLL4, hypoxic siEDN1, or hypoxic dual-knockdown fibroblasts. The inset validates NOTCH1 inhibition after siNOTCH1 transfection. **E **Endothelial validation qPCR in HDMECs treated with fibroblast-conditioned media normalized to viable donor-cell input. The endothelial response-gene score represents the mean relative expression of VEGFA, DLL4, HES1, and HEY1. K-scheme groups: K1 = siControl, K2 = siDLL4, K3 = siNOTCH1, K4 = siEDN1. G-scheme groups: G1 = normoxia control, G2 = hypoxia control, G3 = hypoxia + siDLL4, G4 = hypoxia + siEDN1, G5 = hypoxia + dual siDLL4 + siEDN1. Data are presented as mean ± SEM. *p < 0.05, **p < 0.01, ***p < 0.001; ns, not significant

To investigate the interaction between DLL4/NOTCH and EDN1 signaling, targeted knockdown and functional assays were performed under hypoxic conditions (Fig. 9). RT-qPCR analysis showed that silencing DLL4 or NOTCH1 significantly reduced EDN1 mRNA expression relative to siControl (Fig. 9A; K2 vs. K1, *p* < 0.01; K3 vs. K1, *p* < 0.001), while direct EDN1 knockdown produced the strongest reduction (Fig. 9A; K4 vs. K1, *p* < 0.001). Conversely, HES1 expression was markedly decreased after DLL4 or NOTCH1 knockdown (Fig. 9B; both *p* < 0.001), and was modestly but significantly reduced after EDN1 silencing (Fig. 9B; *p* < 0.05), supporting a bidirectional but asymmetric interaction between the two pathways. CSL/RBPJ luciferase reporter analysis further confirmed functional changes in NOTCH transcriptional activity. Hypoxia significantly increased reporter activity compared with normoxia (Fig. 9C; G2 vs. G1, *p* < 0.001). DLL4 knockdown attenuated this response (Fig. 9C; G3 vs. G2, *p* < 0.05), whereas EDN1 knockdown produced a smaller reduction. Dual DLL4/EDN1 knockdown produced the lowest reporter activity among hypoxic knockdown groups and was significantly reduced relative to hypoxic control (Fig. 9C; G5 vs. G2, *p* < 0.001), while the difference between EDN1 knockdown and dual knockdown was not significant. ELISA analysis showed that hypoxia increased secreted EDN1 levels (Fig. 9D; G2 vs. G1, *p* < 0.001), which were reduced by DLL4 knockdown (Fig. 9D; G3 vs. G2, *p* < 0.01) and strongly suppressed by EDN1 or dual knockdown (Fig. 9D; G4/G5 vs. G2, *p* < 0.001). NOTCH1 inhibition was also independently validated in the inset analysis (Fig. 9D; K3 vs. K1, *p* < 0.01). To assess endothelial consequences of fibroblast signaling changes, HDMECs were treated with conditioned media normalized to viable donor-cell input. Conditioned medium from hypoxic control fibroblasts increased the endothelial angiogenic/NOTCH-response gene score compared with normoxic conditioned medium (Fig. 9E; G2 vs. G1, *p* < 0.01). This response was attenuated by conditioned media from DLL4-, EDN1-, or dual-knockdown fibroblasts (Fig. 9E; G3 vs. G2, *p* < 0.01; G4 vs. G2, *p* < 0.05; G5 vs. G2, *p* < 0.05). Together, these results support asymmetric functional coupling between DLL4/NOTCH and EDN1 signaling. DLL4/NOTCH activity was required for full EDN1 expression and secretion, whereas EDN1 silencing produced a weaker reduction in NOTCH-associated readouts. The conditioned-medium findings indicate that EDN1 contributes to the endothelial-supportive fibroblast secretome but do not exclude additional contributions from VEGF or other hypoxia-induced factors.

### Pharmacological inhibition of canonical NOTCH signaling attenuated hypoxia-induced EDN1 expression

**Fig. 10:**
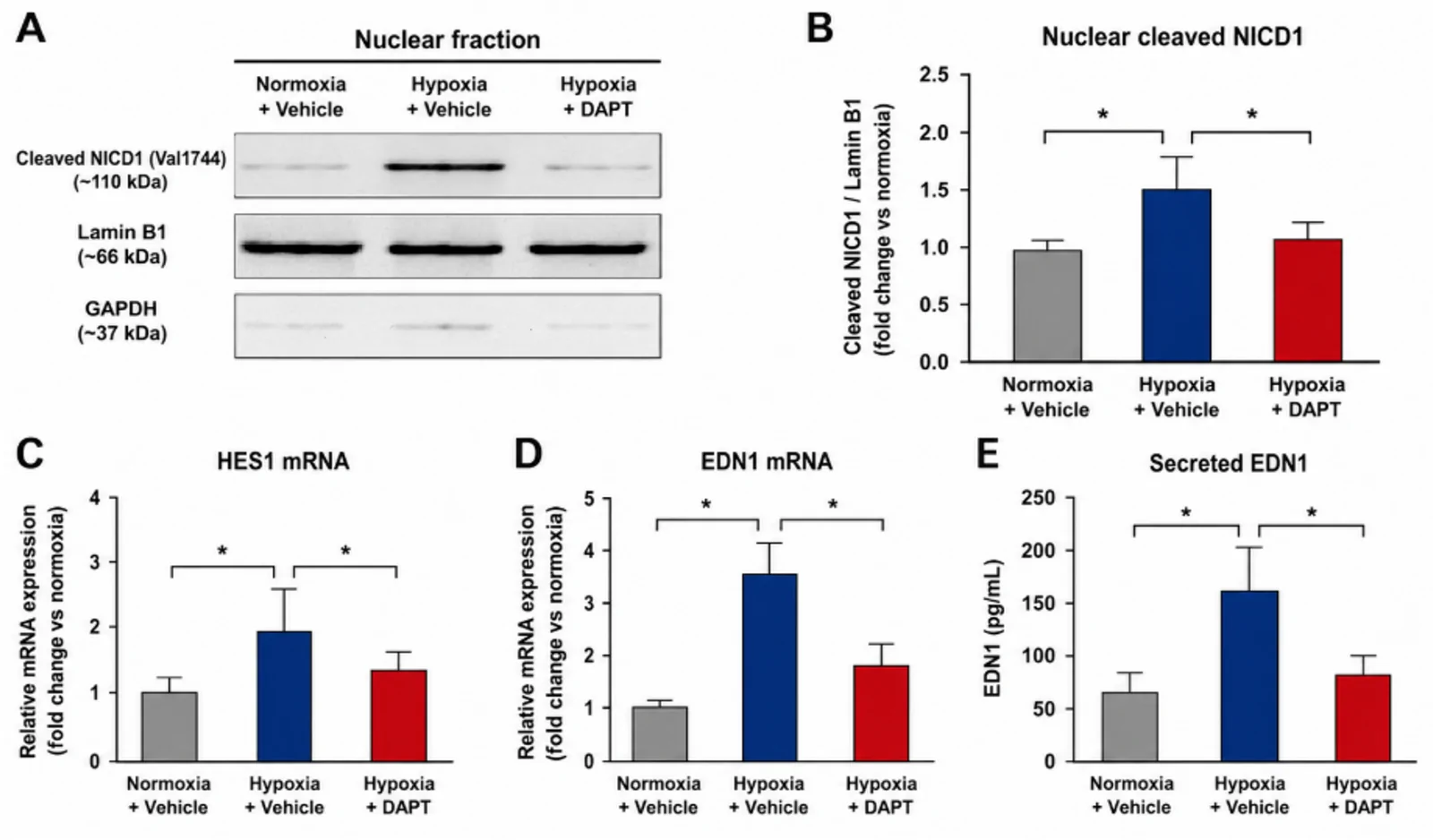
Pharmacological inhibition of canonical NOTCH signaling attenuates hypoxia-induced EDN1 expression. **A **Representative nuclear-fraction Western blot of cleaved NICD1 in HDFs cultured under normoxia, hypoxia, or hypoxia with DAPT treatment. Cleaved NICD1 was detected using a Val1744-specific antibody and appeared at approximately 110 kDa. Lamin B1 (~66 kDa) was used as the nuclear loading control, and GAPDH (~37 kDa) was assessed to exclude cytoplasmic contamination. **B **Densitometric quantification of nuclear cleaved NICD1 normalized to Lamin B1. **C **RT-qPCR analysis of HES1 mRNA expression. **D **RT-qPCR analysis of EDN1 mRNA expression. **E **ELISA quantification of secreted EDN1 in conditioned medium. Data are presented as mean ± SEM from three independent biological replicates. *p < 0.05

To independently assess whether canonical NOTCH activation contributes to hypoxia-induced EDN1 expression, HDFs were treated with DAPT before hypoxic exposure (Fig. 10). Hypoxia significantly increased nuclear cleaved NICD1 abundance compared with normoxia (Fig. 10A–B and Figure S1; *p* < 0.05), whereas DAPT significantly reduced nuclear cleaved NICD1 accumulation relative to the hypoxic vehicle group (*p* < 0.05). Unlike the whole-cell NOTCH1 NTM measurements shown in Figs. 1 and 3, the Val1744-specific antibody used in this experiment detected the approximately 110-kDa cleaved and activated NICD1 form. DAPT also decreased hypoxia-induced HES1 mRNA expression (Fig. 10C; *p* < 0.05), attenuated the increase in EDN1 mRNA (Fig. 10D; *p* < 0.05), and reduced secreted EDN1 levels in conditioned medium (Fig. 10E; *p* < 0.05). These findings provide independent pharmacological evidence that canonical NOTCH activity contributes to full hypoxia-induced EDN1 expression and secretion. However, the experiment does not determine whether this effect reflects direct transcriptional regulation or an indirect pathway-level mechanism.

### Recombinant EDN1 partially rescued DLL4-loss-associated fibroblast, fibrotic, and endothelial-supportive phenotypes

**Fig. 11:**
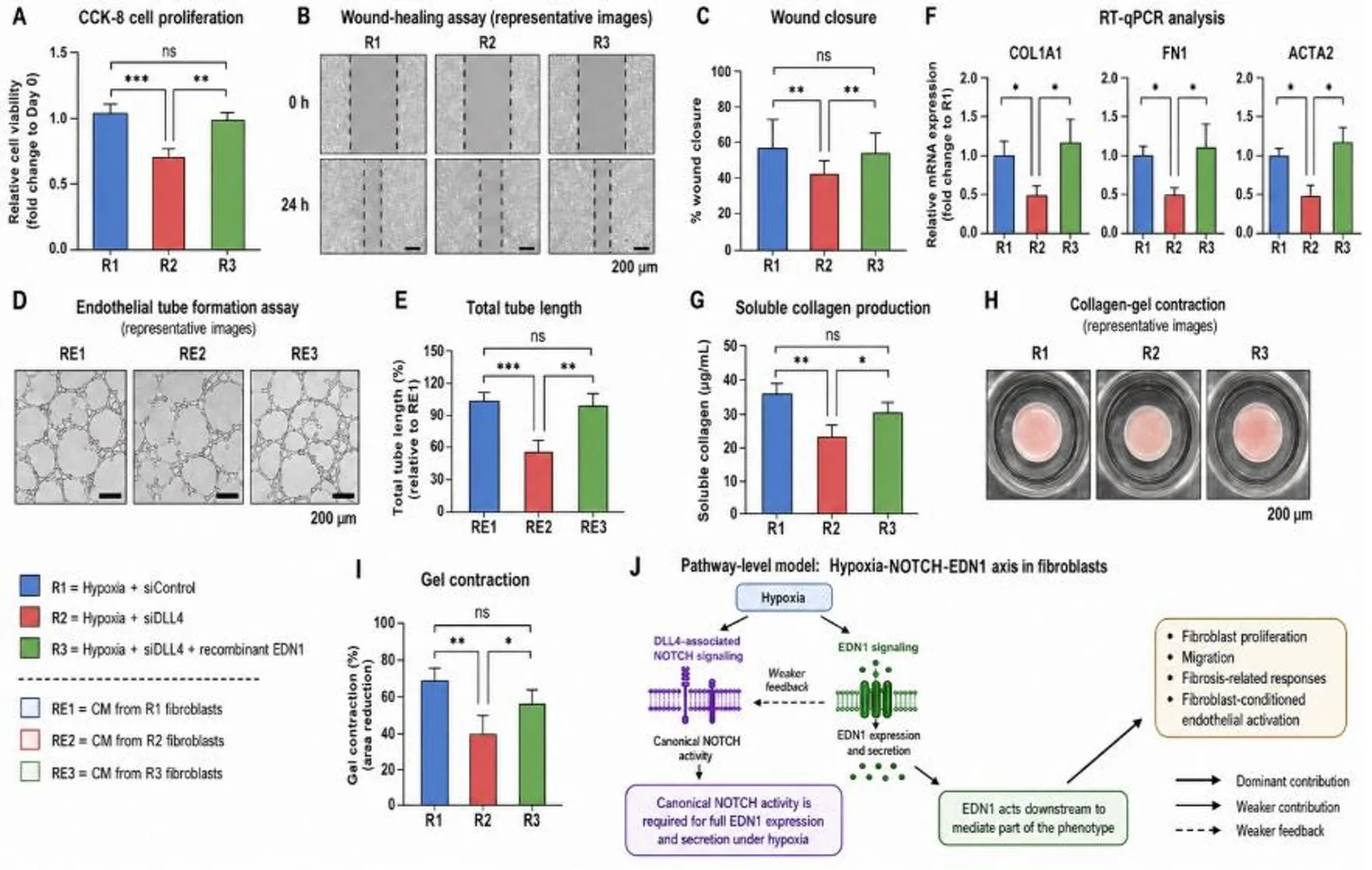
Recombinant EDN1 partially rescues DLL4-loss-associated fibroblast, fibrotic, and endothelial-supportive phenotypes. Rescue groups were defined as R1, hypoxia plus siControl; R2, hypoxia plus siDLL4; and R3, hypoxia plus siDLL4 and recombinant EDN1. Endothelial groups were designated RE1, HDMECs treated with conditioned medium from R1 fibroblasts; RE2, HDMECs treated with conditioned medium from R2 fibroblasts; and RE3, HDMECs treated with conditioned medium from R3 fibroblasts. **A **CCK-8 analysis of fibroblast proliferation. **B **Representative wound-healing images. **C** Quantification of wound closure. **D** Representative endothelial tube-formation images following treatment with fibroblast-conditioned media. **E **Quantification of total tube length. **F **RT-qPCR analysis of COL1A1, FN1, and ACTA2 expression. **G **Quantification of soluble collagen production. **H** Representative collagen-gel contraction images. **I **Quantification of gel contraction. **J **Schematic summary of the pathway-level functional relationship between DLL4-associated NOTCH and EDN1 signaling. Data are presented as mean ± SEM from three independent biological replicates. *p < 0.05, **p < 0.01, ***p < 0.001; ns, not significant

To determine whether EDN1 functionally mediated DLL4-associated fibroblast responses, recombinant EDN1 rescue experiments were performed under hypoxic conditions. The groups were defined as hypoxia plus siControl (R1), hypoxia plus siDLL4 (R2), and hypoxia plus siDLL4 with recombinant EDN1 (R3). DLL4 knockdown significantly reduced fibroblast proliferation compared with hypoxic siControl cells (R2 vs. R1, *p* < 0.001), whereas recombinant EDN1 significantly restored proliferation in DLL4-deficient fibroblasts (R3 vs. R2, *p* < 0.01; Fig. 11A). DLL4 silencing also reduced wound closure relative to R1 (*p* < 0.01), while recombinant EDN1 significantly improved migration compared with the DLL4-knockdown group (R3 vs. R2, *p* < 0.01; Fig. 11B–C). The influence of EDN1 rescue on the fibroblast-conditioned endothelial response was assessed using conditioned-medium tube-formation assays. Conditioned medium from DLL4-silenced fibroblasts markedly reduced endothelial network formation compared with hypoxic siControl-conditioned medium (Fig. 11D–E; RE2 vs. RE1, *p* < 0.001). Treatment of DLL4-silenced fibroblasts with recombinant EDN1 significantly restored the endothelial-supportive activity of their conditioned medium (RE3 vs. RE2, *p* < 0.01), and RE3 was not significantly different from RE1. These findings support a functional contribution of EDN1 to the conditioned-medium response. However, recombinant EDN1 rescue does not establish EDN1 as the sole or dominant soluble mediator because the conditioned medium may also contain VEGF and other hypoxia-induced factors. DLL4 silencing also significantly reduced COL1A1, FN1, and ACTA2 expression compared with R1, whereas recombinant EDN1 increased all three profibrotic markers relative to R2 (Fig. 11F; all *p* < 0.05). Soluble collagen production was significantly decreased by DLL4 knockdown (R2 vs. R1, *p* < 0.01) and partially restored by recombinant EDN1 treatment (R3 vs. R2, *p* < 0.05; Fig. 11G). Consistently, DLL4-deficient fibroblasts exhibited reduced collagen-gel contraction compared with R1 (*p* < 0.01), whereas recombinant EDN1 significantly increased contractile activity relative to R2 (*p* < 0.05; Fig. 11H–I). Collectively, recombinant EDN1 partially restored fibroblast proliferation, migration, profibrotic gene expression, collagen production, contractile activity, and fibroblast-conditioned endothelial tube formation after DLL4 silencing. These findings support EDN1 as a downstream functional mediator of part of the DLL4-associated NOTCH-dependent phenotype. However, the rescue findings do not establish direct transcriptional regulation of EDN1 by NOTCH. The pathway-level interpretation is summarized in Fig. 11J.

### Public transcriptomic datasets support the relevance of DLL4/NOTCH–EDN1 signaling in human keloid-derived material

**Fig. 12:**
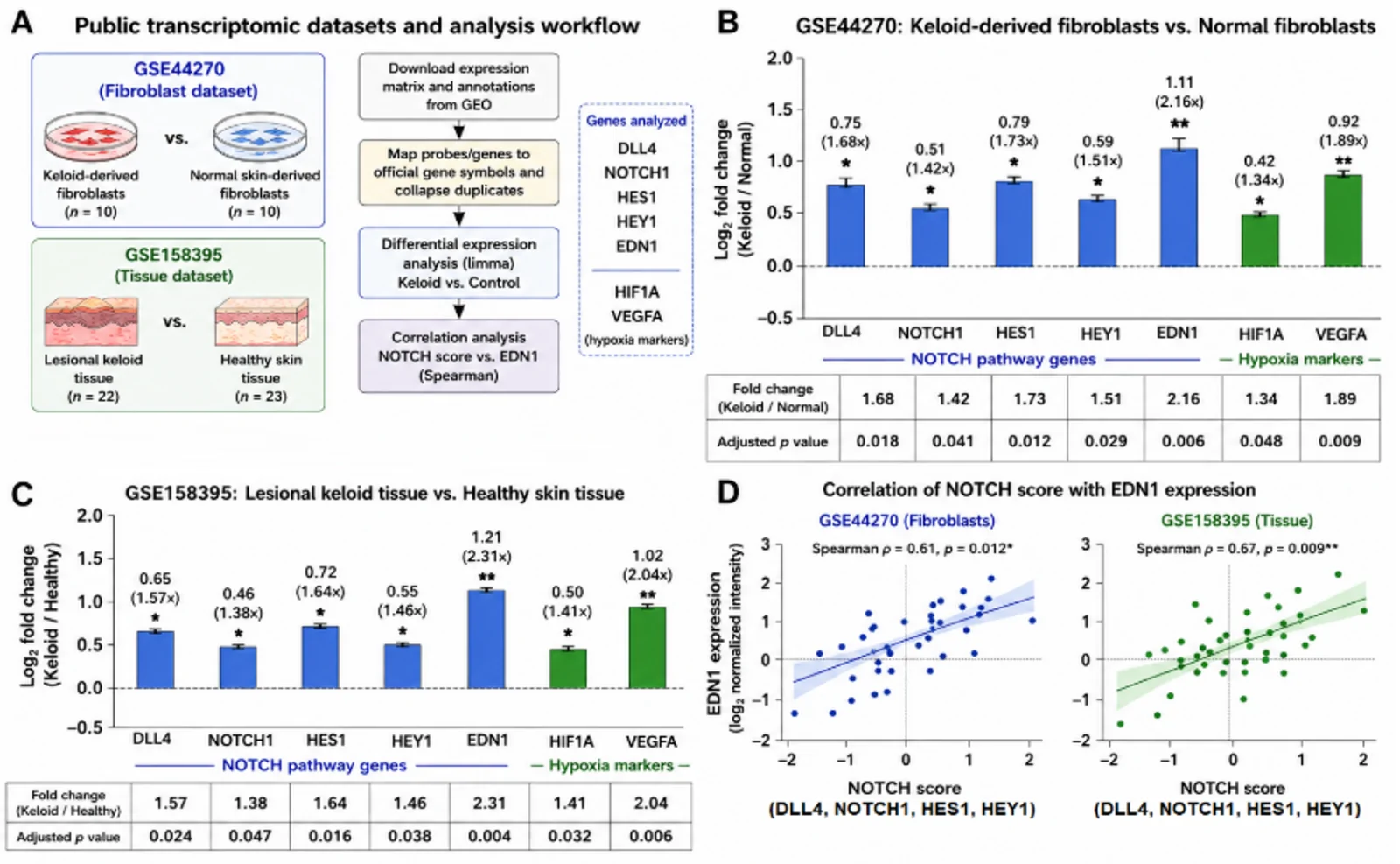
Public transcriptomic validation of DLL4/NOTCH–EDN1 pathway components in human keloid-derived samples. **A **Schematic overview of the datasets and analysis workflow. GSE44270 was used to compare keloid-derived fibroblasts with normal skin-derived fibroblasts, whereas GSE158395 was used to compare lesional keloid tissue with healthy skin. **B **Fold changes in DLL4, NOTCH1, HES1, HEY1, EDN1, HIF1A, and VEGFA expression in keloid-derived fibroblasts relative to normal fibroblasts. **C **Fold changes in the same genes in lesional keloid tissue relative to healthy skin. **D** Spearman correlation between the NOTCH-associated expression score, calculated from DLL4, NOTCH1, HES1, and HEY1, and EDN1 expression in each dataset. Adjusted p values were calculated using the Benjamini–Hochberg method. *p < 0.05, **p < 0.01

To assess the relevance of the experimentally identified signaling relationship in human keloid-derived samples, candidate-gene expression was examined in the GSE44270 fibroblast dataset and the GSE158395 tissue dataset (Fig. 12A). In GSE44270, keloid-derived fibroblasts showed increased expression of several DLL4/NOTCH–EDN1 pathway components compared with normal skin-derived fibroblasts. DLL4 expression increased by 1.68-fold (log₂ fold change = 0.75, adjusted *p* = 0.018), while NOTCH1 increased by 1.42-fold (log₂ fold change = 0.51, adjusted *p* = 0.041). The canonical NOTCH targets HES1 and HEY1 were increased by 1.73-fold (log₂ fold change = 0.79, adjusted *p* = 0.012) and 1.51-fold (log₂ fold change = 0.59, adjusted *p* = 0.029), respectively. EDN1 showed the strongest increase, reaching 2.16-fold relative to normal fibroblasts (log₂ fold change = 1.11, adjusted *p* = 0.006; Fig. 12B). HIF1A and VEGFA were also moderately increased by 1.34-fold (adjusted *p* = 0.048) and 1.89-fold (adjusted *p* = 0.009), respectively. Consistent changes were observed in the GSE158395 tissue dataset. Compared with healthy skin, lesional keloid tissue showed 1.57-fold higher DLL4 expression (log₂ fold change = 0.65, adjusted *p* = 0.024), 1.38-fold higher NOTCH1 expression (log₂ fold change = 0.46, adjusted *p* = 0.047), and 1.64-fold and 1.46-fold increases in HES1 and HEY1, respectively (adjusted *p* = 0.016 and adjusted *p* = 0.038). EDN1 expression was increased by 2.31-fold in lesional keloid tissue (log₂ fold change = 1.21, adjusted *p* = 0.004; Fig. 12C). HIF1A and VEGFA were increased by 1.41-fold and 2.04-fold, respectively (both adjusted *p* < 0.05). The NOTCH-associated expression score was positively correlated with EDN1 expression in both datasets. In GSE44270, the correlation coefficient was ρ = 0.61 (*p* = 0.012), whereas GSE158395 showed a similar positive association (ρ = 0.67, *p* = 0.009; Fig. 12D). These findings indicate coordinated expression of DLL4/NOTCH-related genes and EDN1 in human keloid-derived fibroblasts and tissues. However, the transcriptomic associations do not establish pathway directionality, protein activation, or functional dependency. Together, the public datasets provide supportive human transcriptomic evidence for the potential relevance of the DLL4/NOTCH–EDN1 signaling relationship identified in the normal HDF hypoxia model.

## Discussion

This study investigated how hypoxia-associated signaling may connect fibroblast activation with angiogenic crosstalk in keloid-related remodeling. Consistent with previous evidence that hypoxia activates multiple fibrotic and angiogenic programs, we observed concurrent induction of HIF1A/VEGFA, DLL4-associated NOTCH signaling, and EDN1 signaling in dermal fibroblasts exposed to low oxygen. Therefore, the main implication of this study is not that these pathways are individually hypoxia-responsive, but that DLL4/NOTCH and EDN1 show functional coupling in hypoxic fibroblasts. Specifically, DLL4/NOTCH inhibition reduced EDN1 expression and secretion, EDN1 silencing partially affected NOTCH-associated readouts, dual DLL4/EDN1 knockdown produced stronger suppression of several fibroblast and endothelial-supportive phenotypes, and recombinant EDN1 partially restored the effects lost after DLL4 knockdown. These findings indicate that canonical NOTCH activity is required for full EDN1 expression and secretion under hypoxia and that EDN1 mediates part of the associated fibroblast and endothelial-supportive responses.

Hypoxia is one of the hallmarks of keloid tissue disorders due to excessive deposition of ECM proteins as well as abnormal blood vessel distribution felt to compromise oxygen diffusion [21]. Our results support this observation through strong triggering of hypoxia-dependent gene expression (HIF1A, VEGFA), along with increased activity in NOTCH and endothelin signaling pathways. These observations are also consistent with previous reports indicating that HIF-1α is a direct regulator of both VEGF and Notch ligand expression, thus linking hypoxia sensing to programs involved in angiogenesis and regulating cell fate. In the present study, the concurrent increases in HIF-1α, DLL4, NOTCH1, HES1, HEY1, and EDN1 establish their hypoxia-associated activation but do not demonstrate direct transcriptional cooperation between HIF-1α and NOTCH at the EDN1 locus. Canonical NOTCH activation was supported by increased downstream target expression and nuclear cleaved NICD1, together with their reduction following γ-secretase inhibition. The initial whole-cell immunoblots measured NOTCH1 NTM abundance and were not interpreted as cleavage-specific NICD1 measurements; canonical NOTCH activation was more directly supported by the detection of cleaved NICD1 in nuclear fractions and its reduction following γ-secretase inhibition. Although the role of Notch signaling in endothelial tip versus stalk cell differentiation has been well established [22], its functional relevance to fibroblast development has remained less clearly defined. The results presented herein expand upon this paradigm by illustrating a context-specific contribution of hypoxia-mediated activation of Notch signaling in fibroblasts to fibrosis-associated characteristics, rather than being confined exclusively to endothelial cells.

Loss-of-function analyses indicate that DLL4-associated NOTCH signaling is required to maintain fibroblast proliferation, survival, and cell-cycle progression during hypoxia, and that NOTCH1 contributes functionally to this response. DLL4 or Notch1 knockdown showed decreased proliferation, increased cell death and decreased S phase entry indicating that Notch helps fibroblasts to proliferate during times of stress. Similar to other studies demonstrating that Notch regulates cell cycle and cell survival in different cell types through HES1, cyclin D1, MYC [23, 24]. In addition to its effects on fibroblast proliferation, DLL4/Notch controls fibrotic remodeling, as demonstrated by decreases in relative expression of COL1A1, FN1, ACTA2, decreases in collagen deposition and decreases in contractility with pathway inhibition. This supports data suggesting that Notch promotes myofibroblast differentiation and ECM deposition in multiple fibrotic disease processes (pulmonary and cardiac fibrosis) [25, 26]. Based on these data, hypoxia-associated NOTCH signaling may contribute to maintenance of a myofibroblast-like phenotype in this HDF model; however, the relative contributions of individual NOTCH receptors remain unresolved.

While EDN1 and the endothelin system have been implicated in fibrosis and Vascular dysfunction, their roles in keloid fibroblasts have yet to be fully elucidated [27, 28]. Herein, we provide evidence of hypoxia-mediated induction of EDN1 and its role in mediating fibroblast proliferation, migration, ECM production, and contractility. The effects observed after knockdown of EDN1 phenocopied many (but to a lesser degree) of the effects observed after inhibition of DLL4, suggesting that EDN1 functions primarily as a downstream effector of DLL4 rather than as a primary driver. EDN1 has been linked to MAPK/ERK, PI3K/AKT, and TGF-β signaling in other fibrotic contexts [29, 30], however, these downstream pathways were not directly examined in the present study. The smaller but consistent effect of EDN1 knockdown indicates that EDN1 contributes to, but does not fully account for, the hypoxia-associated fibroblast phenotype. An important finding of this study is the demonstration of functional crosstalk between DLL4/NOTCH and EDN1 signaling. DLL4 or NOTCH1 knockdown significantly decreased EDN1 expression, whereas EDN1 silencing modestly reduced the NOTCH target HES1, supporting asymmetric bidirectional crosstalk. These results identify NOTCH1 as a functionally relevant receptor but do not exclude participation of other NOTCH receptors. Together, the knockdown, DAPT-inhibition, ELISA, reporter, and rescue findings indicate that DLL4/NOTCH signaling acts upstream of EDN1, while EDN1 exerts a weaker feedback effect on NOTCH activity, supporting quantitative asymmetry within the bidirectional signaling relationship.

The practical value of this finding lies in distinguishing pathway activation from pathway dependency. Hypoxic induction of DLL4/NOTCH and EDN1 alone would not establish a meaningful regulatory relationship, because both pathways may be expected to respond to hypoxic stress. However, the knockdown and rescue data suggest that EDN1 is not merely a parallel hypoxia-induced marker, but contributes functionally to DLL4-associated fibroblast proliferation, migration, extracellular matrix remodeling, and paracrine endothelial activation. This functional relationship may have translational relevance, although the endothelial effects of manipulating DLL4/NOTCH–EDN1 signaling must be evaluated in the context of VEGF and other secreted mediators. Further preclinical studies are required before the relative therapeutic value of targeting these pathways can be determined. Thus, identifying EDN1 as a functional mediator of DLL4/NOTCH-associated hypoxic fibroblast activation may help refine therapeutic strategies aimed at modulating both fibrosis and abnormal angiogenic crosstalk in keloid lesions.

The CSL/RBPJ reporter assays further showed that DLL4 knockdown markedly reduced canonical NOTCH activity, whereas EDN1 knockdown produced a more modest reduction. Together with the decrease in HES1 following EDN1 silencing, these findings support pathway-level feedback from EDN1 to NOTCH-associated signaling. The molecular basis of this feedback was not examined. Involvement of shared downstream pathways such as MAPK or TGF-β remains a speculative possibility and will require direct testing using pathway-specific inhibition, phosphorylation analyses, or rescue experiments. Therefore, the present data should not be interpreted as evidence for a defined MAPK- or TGF-β-mediated feedback loop. Likewise, the study did not assess HIF-1α or NICD1/RBPJ occupancy at the EDN1 promoter, promoter-site mutagenesis, or formation of an HIF–NOTCH transcriptional complex; therefore, any direct HIF–NOTCH convergence on EDN1 transcription remains hypothetical.

Because the functional experiments in this study were primarily based on loss-of-function approaches, the conclusions have been revised to reflect pathway requirement rather than direct phenotypic enhancement. Silencing DLL4, NOTCH1, or EDN1 reduced hypoxia-associated fibroblast proliferation, survival, migration, invasion, extracellular matrix remodeling, and endothelial-supportive activity. Therefore, these findings indicate that DLL4/NOTCH and EDN1 signaling are required to maintain these hypoxia-associated phenotypes. Although combined DLL4/EDN1 knockdown produced the strongest suppression in several assays, formal synergy or additivity analysis was not performed. Accordingly, we interpret these results as combined pathway dependency within an asymmetric bidirectional DLL4/NOTCH–EDN1 axis, rather than as cooperative or synergistic enhancement.

Although DLL4 can signal through multiple NOTCH receptors, only NOTCH1 was examined in the present study. The reductions in HES1 activity, EDN1 expression, and EDN1 secretion following NOTCH1 silencing demonstrate that NOTCH1 is a functionally relevant component of the hypoxic response. However, these data do not establish that DLL4-associated effects are mediated exclusively by NOTCH1, because NOTCH2, NOTCH3, and NOTCH4 were not evaluated. In particular, a contribution from NOTCH3 remains possible in fibroblasts. Accordingly, the present findings are interpreted as DLL4-associated NOTCH signaling with an experimentally demonstrated NOTCH1 contribution. Comparative receptor profiling and receptor-specific loss-of-function studies will be required to determine the relative involvement of individual NOTCH receptors.

The conditioned-medium experiments showed that hypoxia-conditioned HDFs altered HDMEC tube formation and migration through changes in the fibroblast secretome. Silencing DLL4 or EDN1 in donor fibroblasts attenuated these endothelial responses, indicating that fibroblast DLL4/NOTCH and EDN1 signaling contributed to the generation of an endothelial-supportive conditioned medium. DLL4 was not evaluated as a soluble mediator; rather, DLL4-associated NOTCH signaling was examined as a fibroblast-intrinsic pathway capable of modifying secreted signals. EDN1 secretion was directly measured and was reduced following DLL4 or NOTCH1 inhibition, while recombinant EDN1 partially restored endothelial tube formation after DLL4 silencing. Nevertheless, the present experiments do not establish EDN1 as the sole or principal mediator of the endothelial response. Hypoxia strongly increased fibroblast VEGFA expression and the endothelial phenotypes may therefore reflect the combined activity of EDN1, VEGF, and other hypoxia-induced soluble factors. Accordingly, the findings demonstrate altered fibroblast–endothelial paracrine communication but do not define the relative contribution of each secreted mediator. Furthermore, the results in this study demonstrate that conditioned media from hypoxic fibroblasts stimulate endothelial sprouting and tube formation, and that both of these effects are significantly reduced following knockdown of either DLL4 or EDN1. The overall conclusion from this study, and others; is that fibroblasts are instrumental in modulating the angiogenic response of endothelial cells to hypoxic conditions, through their secretion of signals that influence the endothelial cell response [31, 32]. Of particular interest is EDN1, a powerful regulator of vascular tone and the behaviour of endothelial cells, promoting their proliferation, migration, and, importantly, tube-forming capacity [33]. DLL4/NOTCH signaling also plays an important role in the process of vascular patterns and the sprouting of new endothelial cells [34]. Our findings suggest that fibroblast DLL4-associated NOTCH signaling may influence endothelial behavior indirectly through regulation of EDN1 secretion. NOTCH1 contributed to this response in the present model, although potential involvement of other NOTCH receptors cannot be excluded.

Rescue experiments showed that recombinant EDN1 partially restored fibroblast proliferation, migration, and endothelial-supportive activity after DLL4 silencing, supporting EDN1 as a downstream functional mediator of DLL4-associated NOTCH signaling. Thus, the rescue data support EDN1 as a downstream functional mediator of DLL4-associated NOTCH signaling. These experiments do not determine which NOTCH receptor or combination of receptors accounts for all DLL4-dependent effects. Moreover, recombinant EDN1 partially restored fibroblast proliferation and migration and fibroblast-conditioned endothelial tube formation after DLL4 silencing, supporting EDN1 as a downstream functional mediator of DLL4-associated NOTCH signaling. EDN1 may therefore represent a potential therapeutic target that warrants further preclinical evaluation in keloid-derived and in vivo models. Collectively, the data support a pathway-level model in which hypoxia activates canonical NOTCH and EDN1 signaling, while genetic and pharmacological inhibition indicate that NOTCH activity is required for full EDN1 expression and secretion. EDN1, in turn, contributes to fibroblast activation and fibroblast-conditioned endothelial responses. The present experiments do not establish direct HIF-1α/NOTCH transcriptional cooperation at the EDN1 promoter or define the molecular intermediates underlying EDN1-to-NOTCH feedback.

Therapies to inhibit both fibroblast activation and aberrant angiogenesis could be developed by targeting this pathway. Notch inhibitors (γ-secretase inhibitors) have shown success in preclinical studies, but there is concern regarding interpersonal toxic side effects when administered systemically [35]. In contrast, using endothelin receptor antagonists that are already approved for the treatment of pulmonary hypertension to inhibit EDN1 signaling may represent a more reasonable option for therapeutic development [29]. The present in vitro findings suggest that NOTCH and EDN1 signaling warrant further evaluation as candidate intervention points in keloid-derived models; they do not establish therapeutic efficacy or patient-level benefit.

An important limitation is that the present experiments were performed in normal human dermal fibroblasts exposed to hypoxia, with TGF-β1 used to establish a profibrotic phenotype, rather than in primary keloid-derived fibroblasts or keloid tissue. Therefore, the findings should be interpreted as mechanistic evidence from a reductionist in vitro model, not as direct proof that the same DLL4/NOTCH–EDN1 interaction operates in keloid lesions in vivo. Nevertheless, this model is relevant because hypoxia and TGF-β1-associated fibroblast activation are central features of fibrotic scar remodeling, and the controlled HDF system allowed us to define pathway dependency and fibroblast–endothelial crosstalk under defined experimental conditions. Future validation in primary keloid fibroblasts, keloid tissue sections, and more complex three-dimensional or co-culture models will be required to confirm disease-specific relevance.

## Conclusion

In conclusion, hypoxia activated DLL4-associated NOTCH, EDN1, and VEGFA-related responses in normal HDFs. Genetic and pharmacological experiments showed that DLL4/NOTCH activity was required for full EDN1 expression and secretion, while EDN1 contributed to fibroblast proliferation, migration, fibrotic remodeling, and contractile activity. Conditioned medium from hypoxic fibroblasts enhanced endothelial migration and tube formation, and these responses were attenuated after DLL4 or EDN1 silencing in donor fibroblasts. However, the relative contributions of EDN1, VEGF, and other hypoxia-induced secreted factors were not determined. The endothelial findings should therefore be interpreted as evidence of altered fibroblast–endothelial paracrine communication rather than identification of a single dominant angiogenic mediator. Direct validation in keloid-derived systems and further characterization of the fibroblast secretome remain necessary.

## Supplementary Information

Supplementary Material 1.

## Data Availability

Any type of the data will be provided by the corresponding author.
