# Supplementary material for: Hypoxia-activated DLL4/NOTCH–EDN1 signaling maintains fibroblast activation and endothelial crosstalk in a human dermal fibroblast model of keloid-like remodeling
Source: Hereditas. 2026 Jul 29;163:119. doi: 10.1186/s41065-026-00717-3 (PMC13700241; doi:10.1186/s41065-026-00717-3)
Supplement: Supplementary file 1 [file 41065_2026_717_MOESM1_ESM.pdf]

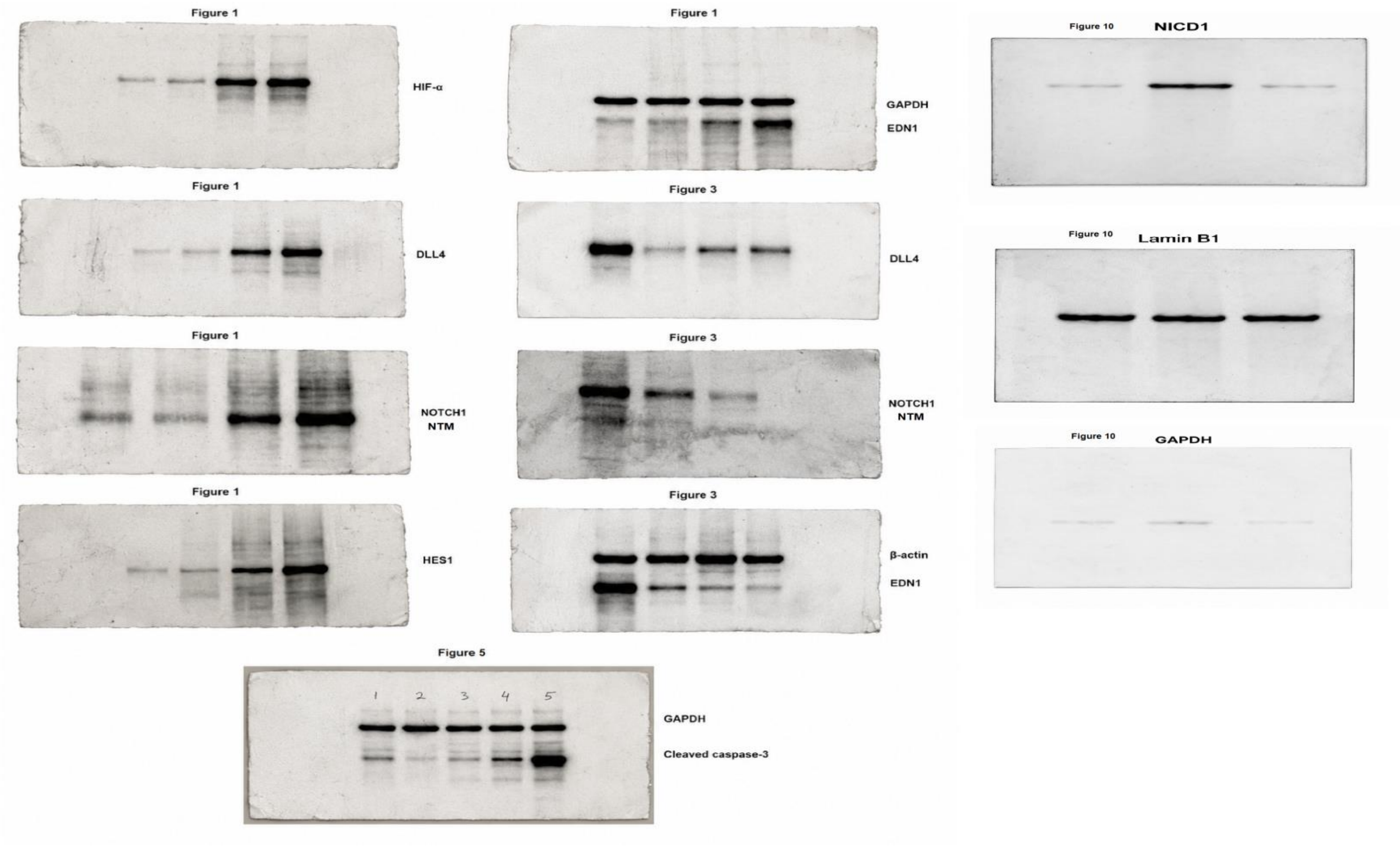

Figure S1: Uncrop Western blot membranes.
